# Genome-wide identification and in silico analysis of *NPF, NRT2, CLC* and *SLAC1/SLAH* nitrate transporters in hexaploid wheat (*Triticum aestivum*)

**DOI:** 10.1038/s41598-022-15202-w

**Published:** 2022-07-03

**Authors:** Aman Kumar, Nitika Sandhu, Pankaj Kumar, Gomsie Pruthi, Jasneet Singh, Satinder Kaur, Parveen Chhuneja

**Affiliations:** School of Agricultural Biotechnology, Punjab Agricultural University, Ludhiana, Punjab India

**Keywords:** Genetics, Molecular biology, Plant sciences, Structural biology

## Abstract

Nitrogen transport is one of the most important processes in plants mediated by specialized transmembrane proteins. Plants have two main systems for nitrogen uptake from soil and its transport within the system—a low-affinity transport system and a high-affinity transport system. Nitrate transporters are of special interest in cereal crops because large amount of money is spent on N fertilizers every year to enhance the crop productivity. Till date four gene families of nitrate transporter proteins; *NPF* (nitrate transporter 1/peptide transporter family), *NRT2* (nitrate transporter 2 family), the *CLC* (chloride channel family), and the *SLAC/SLAH* (slow anion channel-associated homologues) have been reported in plants. In our study, in silico mining of nitrate transporter genes along with their detailed structure, phylogenetic and expression analysis was carried out. A total of 412 nitrate transporter genes were identified in hexaploid wheat genome using HMMER based homology searches in IWGSC Refseq v2.0. Out of those twenty genes were root specific, 11 leaf/shoot specific and 17 genes were grain/spike specific. The identification of nitrate transporter genes in the close proximity to the previously identified 67 marker-traits associations associated with the nitrogen use efficiency related traits in nested synthetic hexaploid wheat introgression library indicated the robustness of the reported transporter genes. The detailed crosstalk between the genome and proteome and the validation of identified putative candidate genes through expression and gene editing studies may lay down the foundation to improve nitrogen use efficiency of cereal crops.

## Introduction

Nitrogen is one of the essential elements required by plants. It is a constituent of nucleic acids, amino acids and proteins and therefore is of great importance in plant physiology and metabolic processes. Though N_2_ is abundant in atmosphere, only legumes are able to fix atmospheric N_2_ with the help of *Rhizobium* bacteria. All other plants mainly absorb N in the form of inorganic ions (ammonium (NH_4_^+^) and nitrate (NO_3_^−^)) from soil. Nitrate is mostly absorbed in aerobic soils, while ammonium is mostly absorbed in acidic soils and wet lands. After uptake, NO_3_^−^ and NH_4_^+^ are assimilated, transformed and mobilized through various processes within plant system.

The agricultural systems focussed on the high-yield crop production remove nitrogen from the soil and depends mostly on the application of large quantities of nitrogenous fertilizers such as urea for the sustained productivity over time. Unfortunately, a large fraction of the applied nitrogen is not directly absorbed by the plants and is lost by the leaching^1^. Despite significant efforts made by the scientific community in the last 50 years, the nitrogen-use efficiency for the cereal crops has not been improved^2^. Beyond this, the economic losses and detrimental environmental consequences caused by the use of large quantities of fertilizers in agriculture are critical issues to be considered^3,4^. Unravelling the genomic regions or the putative candidate genes improving nitrogen-use efficiency will be the first step toward developing nutrient-efficient crop varieties.

To transport N from soil to roots and to other parts of plants, plasma membrane localized proteins known as transporters are essential. They are involved in regulation of N root uptake, root to shoot and leaf to sink transport^5,6^. Plants have evolved two systems for N uptake to cope with changes in N availability. These two systems are the low-affinity transport system (LATS) and high-affinity transport system (HATS). A low-affinity transport system (LATS) is involved where adequate amounts of nitrogen levels are present. A high-affinity transport system (HATS) is involved where limited amounts of N are present. Plants have two low-affinity and two high affinity N transport systems,for nitrate (*NRT1*- low-affinity NO_3_^−^ transporters and *NRT2-*high-affinity NO_3_^−^ transporters) and ammonium (*AMT1*-low-affinity NH_4_^+^ transporters and *AMT2*-high affinity NH_4_^+^ transporters). Majority of N in cereal crops such as wheat is taken up in form of nitrate (NO_3_^−^). Therefore, nitrate transporters are of great importance.

In plants four families of NO_3_^-^ transporters have been identified named *NPF* (*NRT1/PTR*), *NRT2, CLC* (chloride channel) and *SLAC1/SLAH* (slow type anion channel associated homologs)^7^. *NRT1.1* was first NO_3_^-^ transporter to be identified in *Arabidopsis*^8^. The *NRT1* transporter family which has been renamed as *NPF* family is the largest family of nitrate transporters and can further be classified into eight subfamilies^9^. In *Arabidopsis NPF* transporters have been well characterized and contain 53 members divided into eight subfamilies^9^. In rice (*Oryza sativa*) NPF transporters contain 93 members^10^. The majority of NPF transporters are involved in LATS with few exceptions of *NRT1.1/NPF6.3* in *Arabidopsis* and *MtNRT1.3* in *Medicago truncatula*, which are involved in both HATS and LATS^11,12^*.* Although majority of NPFs are involved in nitrate transport, several studies have suggested their role in transport of other substrates such as nitrite^13^, peptides^14^, amino acids^15^ and several plant hormones^16–20^. The second family known as *NRT2* contains high affinity nitrate transporters. A total of seven *NRT2* transporters in *Arabidopsis*^21^ and five *NRT2* transporters in rice have been reported^22,23^. Most of NRT2 transporters require a partner protein—*NAR2* (nitrate assimilation related protein) to function as high affinity nitrate transporters^22–25^. Third family of nitrate transporters, *CLC* (chloride channel) family is mainly associated with vacoular transport of NO_3_^−^^26^. In *Arabidopsis*, six *CLC* genes have been reported and are responsible for nitrate and chloride homoeostasis, thereby regulating stomatal movement and salt tolerance^26–28^. The fourth family—*SLAC1/SLAH* (slow type anion channel associated homologs) is anion channel family. In *Arabidopsis* this family contains four members-*SLAC1*, *SLAH1*, *SLAH2* and *SLAH3* which are involved in the nitrate transport in guard cells and roots and in chloride acquisition^29^. Together these four transporter families are involved in efficient nitrate uptake and utilization in plants.

To the best of our knowledge, the nitrate transporters in hexaploid wheat have not been characterized and explored completely. There are some studies conducted to access the effect of different nitrogen conditions on some of *NPF* and *NRT2* genes^30^. Most of the studies in wheat have been conducted on members of *TaNRT2* gene family. Overexpression of *TaNRT2.5* has been associated with increased grain nitrate uptake and yield^31^. *TaNRT2.1* has been associated with post flowering nitrate uptake in wheat^32^. Expression of *TaNRT2.1* can be induced by nitrogen starvation and abscisic acid (ABA)^33–37^. Some phylogenetic studies and expression-based studies have been conducted on NPF and *NRT2* genes recently^34–36,38^ but *CLC* and *SLAC1/SLAH* genes still remain uncharacterized. Structure of proteins play very important role in the functionality of transporter proteins but still no studies have been conducted on structure prediction of any of *NPF, NRT2, CLC* and *SLAC1/SLAH* genes in wheat. In our study we have identified and characterized genes belonging to all the four families of nitrate transporters. Our analysis includes gene composition, chromosomal location, phylogenetic relations with members of rice and *Arabidopsis* and expression analysis. We adopted a new nomenclature for identified genes as the earlier nomenclature systems do not include complete information about subgenome and homoeologs. We have classified the genes based on phylogeny and identified homoeologous pairs of the gene. Expression profiles of all the genes were studied for different developmental stages and different tissues. Further the structures of all the members of gene families were investigated.

## Methodology

### Sequence search and annotation of nitrate transporter genes

Two methods were used for the identification of *NRT1, NRT2* genes in wheat. In the first method, the CDD IDs (conserved domain database IDs) specific to *TaNPF, TaCLC*, *TaSLAC/TaSLAH* and *TaNRT2* genes (Table 1) were used as identifiers to retrieve genes from the wheat reference genome (IWGSC RefSeq V2.0) from the Ensembl Plants (https://plants.ensembl.org/index.html). In the second method, protein sequences were downloaded from the NCBI database using Nitrate/Nitrogen transporters, and *NRT* as queries. Incomplete, partial sequences, hypothetical, and predicted protein sequences were filtered out. The downloaded sequences were manually curated to remove duplicate sequences and incomplete sequences. The remaining protein sequences (1687 genes) were aligned using Clustal Omega, and the output Stockholm file was used to create the HMMER profile. The HMMER profile was used to search similar protein sequences in the wheat protein database downloaded from IWGSC. A total of 403 high confidence and 38 low confidence proteins were obtained. Separate searches were performed for *TaCLC* and *TaSLAC1/TaSLAH* genes using the same method. A total of 41 *TaCLC* and 43 *TaSLAC1/TaSLAH* high confidence genes and 10 *TaCLC* and 7 *TaSLAC1/TaSLAH* low confidence genes were obtained. The sequences from both the methods were combined, followed by the removal of low confidence proteins and duplicate sequences, and after manual curation, a final set of 412 genes belonging to all four nitrate transporter families were selected. The same methodology was used to identify sequences for *Triticum dicoccoides* (AABB), *T. turgidum* (AABB), *T. urartu* (AA), and *Aegilops tauschii* (DD) for comparative analysis.

**Table 1:** Summary of nitrate transporter gene numbers in wheat, rice, *Arabidopsis* and wheat progenitors.

Conserved domain (CDD/ Pfam Id)	Gene Family	*Triticum aestivum* (AABBDD) (2n = 42)	*Arabidopsis thaliana* (2n = 10)	*Oryza sativa* (2n = 24)	*Triticum dicoccoides* (AABB) (2n = 28)	*Triticum turgidum* (AABB) (2n = 28)	*Triticum urartu* (AA) (2n = 14)	*Aegilops tauchii* (DD) (2n = 14)
cd17413	*NPF6*	22	3	6	15	15	7	7
cd17414	*NPF4*	33	7	12	23	22	9	9
cd17415	*NPF3*	12	1	5	8	8	3	3
cd17416	*NPF1 &2*	47	17	13	39	32	17	16
cd17417	*NPF5*	97	16	32	73	79	31	29
cd17418	*NPF8*	70	5	16	47	47	20	24
cd17419	*NPF7*	11	3	4	10	8	5	3
Cd17341	*NRT2*	46	7	4	20	20	15	18
PF00654	*CLC*	34	6	7	23	22	10	11
PF03595	*SLAC1/ SLAH*	40	5	8	21	21	12	10
Total		412	71	107	253	239	121	120

### Maximum likelihood phylogeny of nitrate transporter genes

The alignments of *TaNRT1/TaNPF*, *TaCLC*, *TaSLAC1/TaSLAH* and *TaNRT2* sequences were created separately using wheat, rice, and *Arabidopsis* sequences by MAFT (E-INS-I algorithm). The evolutionary history was inferred by using the Maximum Likelihood method and JTT matrix-based model. Initial tree(s) for the heuristic search were obtained automatically by applying Neighbour-Join and BioNJ algorithms to a matrix of pairwise distances estimated using the JTT model and then selecting the topology having superior log-likelihood value. Evolutionary studies were conducted in MEGA X. The consistency of the phylogenetic estimate was evaluated by bootstraps (1000 replicates). The resulting tree was visualized using FIGTREE v.1.4.4 (http://tree.bio.ed.ac.uk/software/figtree/).

### Gene structure prediction and identification of homoeologs

The genomic and CDS sequences of genes were downloaded from the Ensembl plants database. The sequence information was utilized to predict the intron/exon positions by using the GSDS server (Gene Structure Display Server, http://gsds.cbi.pku.edu.cn^39^). Separate phylogenies were generated for members of each subfamily to resolve the relationship between them. The analysis was performed in MEGA X by the method described previously. Homoeologous genes were identified based on the phylogenetic relationship between the members of subfamilies. The information regarding physical positions of genes were obtained from Ensembl Plants database. Genome wide distribution map of nitrate transporter genes was developed by web based online visualization tool PhenoGram (http://visualization.ritchielab.org/phenograms/plot).

### Naming of *TaNPF*,* TaNRT2*, *TaCLC* and *TaSLAC1/SLAH* genes

We adopted the method proposed by Schilling et al.^40^ for the naming of *NRT* genes. The genes were named based on their phylogenetic relationships and subgenome location (A, B, or D). Each gene name started with the abbreviation for the species name *Triticum aestivum* (*Ta*), followed by the most closely related *Arabidopsis* gene name (i.e., *NPF1-NPF8, NRT2*), which was followed by the subgenome identifier (A, B, and D). Putative homoeologs were given identical gene names except for the subgenome identifier (*TaNPF4-A1, TaNPF4-B1* and *TaNPF4-D1*). The genes belonging to the same subfamily in the same subgenome were consecutively numbered (Table 2).

**Table 2:** Grouping and Naming of nitrate transporter genes identified in wheat genome Refeq v2.0.

	IWGSC RefSeq ID				Name			
Triad/Tetrad/Diad/ Singleton	A	B	D	Un	A	B	D	Un
TaNPF1-T1	*TraesCS3A02G304400*	*TraesCS3B02G332100*	*TraesCS3D02G297600*		*TaNPF1-3A1*	*TaNPF1-3B1*	*TaNPF1-3D1*	
TaNPF1-T2	*TraesCS3A02G304500*	*TraesCS3B02G332000*	*TraesCS3D02G297400*		*TaNPF1-3A2*	*TaNPF1-3B2*	*TaNPF1-3D2*	
TaNPF2-T1	*TraesCS2A02G045500*	*TraesCS2B02G057700*	*TraesCS2D02G044200*		*TaNPF2-2A1*	*TaNPF2-2B1*	*TaNPF2-2D1*	
TaNPF2-T2	*TraesCS3A02G418700*	*TraesCS3B02G454000*	*TraesCS3D02G414300*		*TaNPF2-3A1*	*TaNPF2-3B1*	*TaNPF2-3D1*	
TaNPF2-T3	*TraesCS3A02G418800*	*TraesCS3B02G454100*	*TraesCS3D02G414400*		*TaNPF2-3A2*	*TaNPF2-3B2*	*TaNPF2-3D2*	
TaNPF2-T4	*TraesCS4A02G283900*	*TraesCS4B02G029600*	*TraesCS4D02G026800*		*TaNPF2-4A1*	*TaNPF2-4B1*	*TaNPF2-4D1*	
TaNPF2-S1	*TraesCS4A02G440300*				*TaNPF2-4A2*			
TaNPF2-S2	*TraesCS4A02G440400*				*TaNPF2-4A3*			
TaNPF2-S3	*TraesCS4A02G440500*				*TaNPF2-4A4*			
TaNPF2-S4	*TraesCS4A02G440600*				*TaNPF2-4A5*			
TaNPF2-S5	*TraesCS4A02G440700*				*TaNPF2-4A6*			
TaNPF2-T5	*TraesCS5A02G004400*	*TraesCS5B02G001100*	*TraesCS5D02G012500*		*TaNPF2-5A1*	*TaNPF2-5B1*	*TaNPF2-5D1*	
TaNPF2-T6	*TraesCS5A02G037900*	*TraesCS5B02G039100*	*TraesCS5D02G045300*		*TaNPF2-5A2*	*TaNPF2-5B2*	*TaNPF2-5D2*	
TaNPF2-T7	*TraesCS5A02G153200*	*TraesCS5B02G152000*	*TraesCS5D02G158500*		*TaNPF2-5A3*	*TaNPF2-5B3*	*TaNPF2-5D3*	
TaNPF2-D1	*TraesCS7A02G054000*		*TraesCS7D02G049300*		*TaNPF2-7A1*		*TaNPF2-7D1*	
TaNPF2-D2	*TraesCS7A02G054100*		*TraesCS7D02G049400*		*TaNPF2-7A2*		*TaNPF2-7D2*	
TaNPF2-T8	*TraesCS7A02G121600*	*TraesCS7B02G020200*	*TraesCS7D02G119800*		*TaNPF2-7A3*	*TaNPF2-7B3*	*TaNPF2-7D3*	
TaNPF2-T9	*TraesCS7A02G121700*	*TraesCS7B02G020500*	*TraesCS7D02G120200*		*TaNPF2-7A4*	*TaNPF2-7B4*	*TaNPF2-7D4*	
TaNPF2-D3		*TraesCS2B02G057600*	*TraesCS2D02G044000*			*TaNPF2-7B5*	*TaNPF2-7D5*	
TaNPF2-D4		*TraesCS7B02G020300*	*TraesCS7D02G119900*			*TaNPF2-7B6*	*TaNPF2-7D6*	
TaNPF2-S6			*TraesCS7D02G076900*				*TaNPF2-7D7*	
TaNPF3-T1	*TraesCS1A02G257400*	*TraesCS1B02G267900*	*TraesCS1D02G256700*		*TaNPF3-1A1*	*TaNPF3-1B1*	*TaNPF3-1D1*	
TaNPF3-T2	*TraesCS1A02G257800*	*TraesCS1B02G268200*	*TraesCS1D02G257100*		*TaNPF3-1A2*	*TaNPF3-1B2*	*TaNPF3-1D2*	
TaNPF3-T3	*TraesCS1A02G257900*	*TraesCS1B02G268300*	*TraesCS1D02G257200*		*TaNPF3-1A3*	*TaNPF3-1B3*	*TaNPF3-1D3*	
TaNPF3-T4	*TraesCS7A02G206400*	*TraesCS7B02G113600*	*TraesCS7D02G209200*		*TaNPF3-7A1*	*TaNPF3-7B1*	*TaNPF3-7D1*	
TaNPF4-T1	*TraesCS2A02G264500*	*TraesCS2B02G277600*	*TraesCS2D02G259400*		*TaNPF4-2A1*	*TaNPF4-2B1*	*TaNPF4-2D1*	
TaNPF4-T2	*TraesCS2A02G309100*	*TraesCS2B02G326200*	*TraesCS2D02G307400*		*TaNPF4-2A2*	*TaNPF4-2B2*	*TaNPF4-2D2*	
TaNPF4-T3	*TraesCS2A02G350000*	*TraesCS2B02G368500*	*TraesCS2D02G348400*		*TaNPF4-2A3*	*TaNPF4-2B3*	*TaNPF4-2D3*	
TaNPF4-T4	*TraesCS2A02G350100*	*TraesCS2B02G368600*	*TraesCS2D02G348500*		*TaNPF4-2A4*	*TaNPF4-2B4*	*TaNPF4-2D4*	
TaNPF4-D1	*TraesCS2A02G350200*	*TraesCS2B02G368400*			*TaNPF4-2A5*	*TaNPF4-2B5*		
TaNPF4-T5	*TraesCS2A02G350300*	*TraesCS2B02G368700*	*TraesCS2D02G348600*		*TaNPF4-2A6*	*TaNPF4-2B6*	*TaNPF4-2D6*	
TaNPF4-S1	*TraesCS3A02G272600*				*TaNPF4-3A1*			
TaNPF4-T6	*TraesCS4A02G225400*	*TraesCS4B02G090800*	*TraesCS4D02G087900*		*TaNPF4-4A1*	*TaNPF4-4B1*	*TaNPF4-4D1*	
TaNPF4-T7	*TraesCS5A02G056100*	*TraesCS5B02G060800*	*TraesCS5D02G067100*		*TaNPF4-5A1*	*TaNPF4-5B1*	*TaNPF4-5D1*	
TaNPF4-T8	*TraesCS5A02G056200*	*TraesCS5B02G060500*	*TraesCS5D02G067400*		*TaNPF4-5A2*	*TaNPF4-5B2*	*TaNPF4-5D2*	
TaNPF4-T9	*TraesCS5A02G388000*	*TraesCS5B02G393100*	*TraesCS5D02G398000*		*TaNPF4-5A3*	*TaNPF4-5B3*	*TaNPF4-5D3*	
TaNPF4-T10	*TraesCS7A02G365100*	*TraesCS7B02G262200*	*TraesCS7D02G357300*		*TaNPF4-7A1*	*TaNPF4-7B1*	*TaNPF4-7D1*	
TaNPF5-T1	*TraesCS1A02G150200*	*TraesCS1B02G168000*	*TraesCS1D02G147200*		*TaNPF5-1A1*	*TaNPF5-1B1*	*TaNPF5-1D1*	
TaNPF5-T2	*TraesCS1A02G150400*	*TraesCS1B02G168100*	*TraesCS1D02G147400*		*TaNPF5-1A2*	*TaNPF5-1B2*	*TaNPF5-1D2*	
TaNPF5-T3	*TraesCS1A02G269400*	*TraesCS1B02G279900*	*TraesCS1D02G269500*		*TaNPF5-1A3*	*TaNPF5-1B3*	*TaNPF5-1D3*	
TaNPF5-T4	*TraesCS1A02G269500*	*TraesCS1B02G280000*	*TraesCS1D02G269600*		*TaNPF5-1A4*	*TaNPF5-1B4*	*TaNPF5-1D4*	
TaNPF5-T5	*TraesCS1A02G269600*	*TraesCS1B02G280100*	*TraesCS1D02G269700*		*TaNPF5-1A5*	*TaNPF5-1B5*	*TaNPF5-1D5*	
TaNPF5-T6	*TraesCS2A02G565600*	*TraesCS2B02G626000*	*TraesCS2D02G576000*		*TaNPF5-2A1*	*TaNPF5-2B1*	*TaNPF5-2D1*	
TaNPF5-D1	*TraesCS2A02G571800*		*TraesCS2D02G583300*		*TaNPF5-2A2*		*TaNPF5-2D2*	
TaNPF5-T7	*TraesCS2A02G571900*	*TraesCS2B02G615500*	*TraesCS2D02G583400*		*TaNPF5-2A3*	*TaNPF5-2B3*	*TaNPF5-2D3*	
TaNPF5-D2	*TraesCS2A02G572000*	*TraesCS2B02G615400*			*TaNPF5-2A4*	*TaNPF5-2B4*		
TaNPF5-S1	*TraesCS2A02G572100*				*TaNPF5-2A5*			
TaNPF5-T8	*TraesCS2A02G572200*	*TraesCS2B02G615300*	*TraesCS2D02G583500*		*TaNPF5-2A6*	*TaNPF5-2B6*	*TaNPF5-2D6*	
TaNPF5-T9	*TraesCS2A02G572300*	*TraesCS2B02G615200*	*TraesCS2D02G583600*		*TaNPF5-2A7*	*TaNPF5-2B7*	*TaNPF5-2D7*	
TaNPF5-T10	*TraesCS3A02G185600*	*TraesCS3B02G215200*	*TraesCS3D02G189500*		*TaNPF5-3A1*	*TaNPF5-3B1*	*TaNPF5-3D1*	
TaNPF5-T11	*TraesCS3A02G382100*	*TraesCS3B02G414800*	*TraesCS3D02G375900*		*TaNPF5-3A2*	*TaNPF5-3B2*	*TaNPF5-3D2*	
TaNPF5-T12	*TraesCS3A02G382200*	*TraesCS3B02G414900*	*TraesCS3D02G375800*		*TaNPF5-3A3*	*TaNPF5-3B3*	*TaNPF5-3D3*	
TaNPF5-T13	*TraesCS3A02G382300*	*TraesCS3B02G415200*	*TraesCS3D02G375700*		*TaNPF5-3A4*	*TaNPF5-3B4*	*TaNPF5-3D4*	
TaNPF5-T14	*TraesCS3A02G382400*	*TraesCS3B02G415300*	*TraesCS3D02G375600*		*TaNPF5-3A5*	*TaNPF5-3B5*	*TaNPF5-3D5*	
TaNPF5-D3	*TraesCS3A02G382600*		*TraesCS3D02G375500*		*TaNPF5-3A6*		*TaNPF5-3D6*	
TaNPF5-D4	*TraesCS3A02G382700*		*TraesCS3D02G375400*		*TaNPF5-3A7*		*TaNPF5-3D7*	
TaNPF5-D5	*TraesCS3A02G382800*		*TraesCS3D02G375300*		*TaNPF5-3A8*		*TaNPF5-3D8*	
TaNPF5-D6	*TraesCS3A02G382900*		*TraesCS3D02G375200*		*TaNPF5-3A9*		*TaNPF5-3D9*	
TaNPF5-T15	*TraesCS3A02G383200*	*TraesCS3B02G415600*	*TraesCS3D02G376200*		*TaNPF5-3A10*	*TaNPF5-3B10*	*TaNPF5-3D10*	
TaNPF5-T16	*TraesCS3A02G383300*	*TraesCS3B02G415700*	*TraesCS3D02G376300*		*TaNPF5-3A11*	*TaNPF5-3B11*	*TaNPF5-3D11*	
TaNPF5-T17	*TraesCS5A02G485000*	*TraesCS5B02G498400*	*TraesCS5D02G498500*		*TaNPF5-5A1*	*TaNPF5-5B1*	*TaNPF5-5D1*	
TaNPF5-T18	*TraesCS5A02G485200*	*TraesCS5B02G498500*	*TraesCS5D02G498700*		*TaNPF5-5A2*	*TaNPF5-5B2*	*TaNPF5-5D2*	
TaNPF5-T19	*TraesCS5A02G485300*	*TraesCS5B02G498700*	*TraesCS5D02G498800*		*TaNPF5-5A3*	*TaNPF5-5B3*	*TaNPF5-5D3*	
TaNPF5-S2	*TraesCS5A02G508500*				*TaNPF5-5A4*			
TaNPF5-T20	*TraesCS6A02G041300*	*TraesCS6B02G056500*	*TraesCS6D02G047600*		*TaNPF5-6A1*	*TaNPF5-6B1*	*TaNPF5-6D1*	
TaNPF5-T21	*TraesCS7A02G196100*	*TraesCS7B02G101800*	*TraesCS7D02G197600*		*TaNPF5-7A1*	*TaNPF5-7B1*	*TaNPF5-7D1*	
TaNPF5-T22	*TraesCS7A02G461200*	*TraesCS7B02G362700*	*TraesCS7D02G449400*		*TaNPF5-7A2*	*TaNPF5-7B2*	*TaNPF5-7D2*	
TaNPF5-D7	*TraesCS7A02G504300*		*TraesCS7D02G491400*		*TaNPF5-7A3*		*TaNPF5-7D3*	
TaNPF5-S3		*TraesCS2B02G013000*				*TaNPF5-2B8*		
TaNPF5-S4		*TraesCS2B02G248000*				*TaNPF5-2B9*		
TaNPF5-S5		*TraesCS2B02G401000*				*TaNPF5-2B10*		
TaNPF5-S6		*TraesCS2B02G626100*				*TaNPF5-2B11*		
TaNPF5-S7		*TraesCS2B02G626600*				*TaNPF5-2B12*		
TaNPF5-S8		*TraesCS2B02G626700*				*TaNPF5-2B13*		
TaNPF5-S9		*TraesCS3B02G304500*				*TaNPF5-3B12*		
TaNPF5-S10		*TraesCS3B02G415000*				*TaNPF5-3B13*		
TaNPF5-S11		*TraesCS3B02G415100*				*TaNPF5-3B14*		
TaNPF5-S12		*TraesCS4B02G057000*				*TaNPF5-4B1*		
TaNPF5-S13		*TraesCS4B02G338600*				*TaNPF5-4B2*		
TaNPF5-S14			*TraesCS4D02G335100*				*TaNPF5-4D1*	
TaNPF5-D8		*TraesCS7B02G040100*	*TraesCS7D02G139600*			*TaNPF5-7B4*	*TaNPF5-7D4*	
TaNPF5-S15		*TraesCS7B02G312500*				*TaNPF5-7B5*		
TaNPF6-T1	*TraesCS1A02G031300*	*TraesCS1B02G038700*	*TraesCS1D02G032700*		*TaNPF6-1A1*	*TaNPF6-1B1*	*TaNPF6-1D1*	
TaNPF6-T2	*TraesCS1A02G210900*	*TraesCS1B02G224900*	*TraesCS1D02G214200*		*TaNPF6-1A2*	*TaNPF6-1B2*	*TaNPF6-1D2*	
TaNPF6-T3	*TraesCS1A02G211000*	*TraesCS1B02G225000*	*TraesCS1D02G214300*		*TaNPF6-1A3*	*TaNPF6-1B3*	*TaNPF6-1D3*	
TaNPF6-T4	*TraesCS2A02G335800*	*TraesCS2B02G346100*	*TraesCS2D02G327000*		*TaNPF6-2A1*	*TaNPF6-2B1*	*TaNPF6-2D1*	
TaNPF6-S1		*TraesCS4B02G371000*				*TaNPF6-4B1*		
TaNPF6-S2		*TraesCS4B02G375800*				*TaNPF6-4B2*		
TaNPF6-S3			*TraesCS4D02G361500*				*TaNPF6-4D3*	
TaNPF6-T5	*TraesCS5A02G409600*	*TraesCS5B02G414000*	*TraesCS5D02G419200*		*TaNPF6-5A1*	*TaNPF6-5B1*	*TaNPF6-5D1*	
TaNPF6-S4	*TraesCS5A02G537100*				*TaNPF6-5A2*			
TaNPF6-T6	*TraesCS7A02G301700*	*TraesCS7B02G201900*	*TraesCS7D02G297000*		*TaNPF6-7A1*	*TaNPF6-7B1*	*TaNPF6-7D1*	
TaNPF7-S1	*TraesCS4A02G284300*				*TaNPF7-4A1*			
TaNPF7-S2	*TraesCS5A02G546200*				*TaNPF7-5A1*			
TaNPF7-T1	*TraesCS6A02G263500*	*TraesCS6B02G290500*	*TraesCS6D02G251500*		*TaNPF7-6A1*	*TaNPF7-6B1*	*TaNPF7-6D1*	
TaNPF7-T2	*TraesCS6A02G280200*	*TraesCS6B02G309200*	*TraesCS6D02G260500*		*TaNPF7-6A2*	*TaNPF7-6B2*	*TaNPF7-6D2*	
TaNPF7-S3	*TraesCS7A02G413200*				*TaNPF7-7A1*			
TaNPF7-S4		*TraesCS4B02G380000*				*TaNPF7-4B2*		
TaNPF7-S5				*TraesCSU02G130200*				*TaNPF7-Un1*
TaNPF8-T1	*TraesCS2A02G416800*	*TraesCS2B02G000500*	*TraesCS2D02G413900*		*TaNPF8-2A1*	*TaNPF8-2B1*	*TaNPF8-2D1*	
TaNPF8-T2	*TraesCS3A02G056400*	*TraesCS3B02G069100*	*TraesCS3D02G056300*		*TaNPF8-3A1*	*TaNPF8-3B1*	*TaNPF8-3D1*	
TaNPF8-T3	*TraesCS3A02G057000*	*TraesCS3B02G070200*	*TraesCS3D02G056700*		*TaNPF8-3A2*	*TaNPF8-3B2*	*TaNPF8-3D2*	
TaNPF8-T4	*TraesCS3A02G392800*	*TraesCS3B02G424700*	*TraesCS3D02G385600*		*TaNPF8-3A3*	*TaNPF8-3B3*	*TaNPF8-3D3*	
TaNPF8-T5	*TraesCS3A02G392900*	*TraesCS3B02G424800*	*TraesCS3D02G385700*		*TaNPF8-3A4*	*TaNPF8-3B4*	*TaNPF8-3D4*	
TaNPF8-T6	*TraesCS4A02G075700*	*TraesCS4B02G231500*	*TraesCS4D02G232900*		*TaNPF8-4A1*	*TaNPF8-4B1*	*TaNPF8-4D1*	
TaNPF8-T7	*TraesCS4A02G075900*	*TraesCS4B02G231700*	*TraesCS4D02G233000*		*TaNPF8-4A2*	*TaNPF8-4B2*	*TaNPF8-4D2*	
TaNPF8-T8	*TraesCS4A02G076000*	*TraesCS4B02G231800*	*TraesCS4D02G233100*		*TaNPF8-4A3*	*TaNPF8-4B3*	*TaNPF8-4D3*	
TaNPF8-T9	*TraesCS4A02G076100*	*TraesCS4B02G232000*	*TraesCS4D02G233000*		*TaNPF8-4A4*	*TaNPF8-4B4*	*TaNPF8-4D4*	
TaNPF8-T10	*TraesCS4A02G076200*	*TraesCS4B02G232100*	*TraesCS4D02G233400*		*TaNPF8-4A5*	*TaNPF8-4B5*	*TaNPF8-4D5*	
TaNPF8-T11	*TraesCS4A02G262700*	*TraesCS4B02G052200*	*TraesCS4D02G052400*		*TaNPF8-4A6*	*TaNPF8-4B6*	*TaNPF8-4D6*	
TaNPF8-S1	*TraesCS4A02G287300*				*TaNPF8-4A7*			
TaNPF8-S2	*TraesCS4A02G287900*				*TaNPF8-4A8*			
TaNPF8-T12	*TraesCS6A02G142600*	*TraesCS6B02G171000*	*TraesCS6D02G132100*		*TaNPF8-6A1*	*TaNPF8-6B1*	*TaNPF8-6D1*	
TaNPF8-D1	*TraesCS7A02G095200*		*TraesCS7D02G091600*		*TaNPF8-7A1*		*TaNPF8-7D1*	
TaNPF8-T13	*TraesCS7A02G381500*	*TraesCS7B02G283400*	*TraesCS7D02G377800*		*TaNPF8-7A2*	*TaNPF8-7B2*	*TaNPF8-7D2*	
TaNPF8-S3	*TraesCS7A02G381600*				*TaNPF8-7A3*			
TaNPF8-T14	*TraesCS7A02G381700*	*TraesCS7B02G283800*	*TraesCS7D02G377900*		*TaNPF8-7A4*	*TaNPF8-7B4*	*TaNPF8-7D4*	
TaNPF8-T15	*TraesCS7A02G381800*	*TraesCS7B02G284300*	*TraesCS7D02G378300*		*TaNPF8-7A5*	*TaNPF8-7B5*	*TaNPF8-7D5*	
TaNPF8-D2	*TraesCS7A02G412100*	*TraesCS7B02G311400*			*TaNPF8-7A6*	*TaNPF8-7B6*		
TaNPF8-T16	*TraesCS7A02G413100*	*TraesCS7B02G312600*	*TraesCS7D02G406200*		*TaNPF8-7A7*	*TaNPF8-7B7*	*TaNPF8-7D7*	
TaNPF8-T17	*TraesCS7A02G413300*	*TraesCS7B02G312700*	*TraesCS7D02G406400*		*TaNPF8-7A8*	*TaNPF8-7B8*	*TaNPF8-7D8*	
TaNPF8-S4	*TraesCS7A02G531000*				*TaNPF8-7A9*			
TaNPF8-D3		*TraesCS3B02G069900*	*TraesCS3D02G057000*			*TaNPF8-3B5*	*TaNPF8-3D5*	
TaNPF8-D4		*TraesCS4B02G026700*	*TraesCS4D02G024400*			*TaNPF8-4B9*	*TaNPF8-4D9*	
TaNPF8-S5		*TraesCS4B02G398100*				*TaNPF8-4B10*		
TaNPF8-S6		*TraesCS5B02G245300*				*TaNPF8-5B1*		
TaNPF8-D5		*TraesCS6B02G406100*	*TraesCS6D02G353500*			*TaNPF8-6B2*	*TaNPF8-6D2*	
TaNPF8-S7			*TraesCS7D02G518900*				*TaNPF8-7D9*	
TaNPF8-S8				*TraesCSU02G207500*				*TaNPF8-Un1*
TaNPF8-S9				*TraesCSU02G115500*				*TaNPF8-Un2*
TaNRT2-D1	*TraesCS2A02G074800*		*TraesCS2D02G073500*		*TaNRT2-2A1*		*TaNRT2-2D1*	
TaNRT2-T1	*TraesCS3A02G254000*	*TraesCS3B02G285900*	*TraesCS3D02G254900*		*TaNRT2-3A1*	*TaNRT2-3B1*	*TaNRT2-3D1*	
TaNRT2-D2	*TraesCS6A02G030700*	*TraesCS6B02G044100*			*TaNRT2-6A1*	*TaNRT2-6B1*		
TaNRT2-T2	*TraesCS6A02G030800*	*TraesCS6B02G044400*	*TraesCS6D02G035900*		*TaNRT2-6A2*	*TaNRT2-6B2*	*TaNRT2-6D2*	
TaNRT2-T3	*TraesCS6A02G030900*	*TraesCS6B02G044300*	*TraesCS6D02G035800*		*TaNRT2-6A3*	*TaNRT2-6B3*	*TaNRT2-6D3*	
TaNRT2-T4	*TraesCS6A02G031000*	*TraesCS6B02G044200*	*TraesCS6D02G035700*		*TaNRT2-6A4*	*TaNRT2-6B4*	*TaNRT2-6D4*	
TaNRT2-D3	*TraesCS6A02G031100*	*TraesCS6B02G044500*			*TaNRT2-6A5*	*TaNRT2-6B5*		
TaNRT2-T5	*TraesCS6A02G031200*	*TraesCS6B02G044000*	*TraesCS6D02G035600*		*TaNRT2-6A6*	*TaNRT2-6B6*	*TaNRT2-6D6*	
TaNRT2-T6	*TraesCS6A02G032400*	*TraesCS6B02G045600*	*TraesCS6D02G037200*		*TaNRT2-6A7*	*TaNRT2-6B7*	*TaNRT2-6D7*	
TaNRT2-T7	*TraesCS6A02G032500*	*TraesCS6B02G045700*	*TraesCS6D02G037300*		*TaNRT2-6A8*	*TaNRT2-6B8*	*TaNRT2-6D8*	
TaNRT2-T8	*TraesCS6A02G032800*	*TraesCS6B02G046500*	*TraesCS6D02G037800*		*TaNRT2-6A9*	*TaNRT2-6B9*	*TaNRT2-6D9*	
TaNRT2-D4	*TraesCS6A02G032900*		*TraesCS6D02G037900*		*TaNRT2-6A10*		*TaNRT2-6D10*	
TaNRT2-TT1	*TraesCS6A02G033000*	*TraesCS6B02G046600*	*TraesCS6D02G038100*	*TraesCS6D02G038000*	*TaNRT2-6A11*	*TaNRT2-6B11*	*TaNRT2-6D11x*	*TaNRT2-6D11y*
TaNRT2-D5	*TraesCS6A02G033100*		*TraesCS6D02G038300*		*TaNRT2-6A12*		*TaNRT2-6D12*	
TaNRT2-T9	*TraesCS6A02G033200*	*TraesCS6B02G046700*	*TraesCS6D02G038200*		*TaNRT2-6A13*	*TaNRT2-6B13*	*TaNRT2-6D13*	
TaNRT2-T10	*TraesCS7A02G428500*	*TraesCS7B02G328700*	*TraesCS7D02G420900*		*TaNRT2-7A1*	*TaNRT2-7B1*	*TaNRT2-7D1*	
TaNRT2-D6			*TraesCS1D02G035700*	*TraesCSU02G002800*			*TaNRT2-1D1*	*TaNRT2-Un1*
TaCLC-T1	*TraesCS2A02G309900*	*TraesCS2B02G326900*	*TraesCS2D02G308100*		*TaCLC-2A1*	*TaCLC-2B1*	*TaCLC-2D1*	
TaCLC-T2	*TraesCS2A02G517500*	*TraesCS2B02G546000*	*TraesCS2D02G519000*		*TaCLC-2A2*	*TaCLC-2B2*	*TaCLC-2D2*	
TaCLC-T3	*TraesCS3A02G253600*	*TraesCS3B02G285500*	*TraesCS3D02G254500*		*TaCLC-3A1*	*TaCLC-3B1*	*TaCLC-3D1*	
TaCLC-TT1	*TraesCS3A02G125300*	*TraesCS3B02G144700*	*TraesCS3D02G126700*	*TraesCS3D02G126600*	*TaCLC-3A2*	*TaCLC-3B2*	*TaCLC-3D2x*	*TaCLC-3D2y*
TaCLC-T4	*TraesCS3A02G390100*	*TraesCS3B02G418700*	*TraesCS3D02G379600*		*TaCLC-3A3*	*TaCLC-3B3*	*TaCLC-3D3*	
TaCLC-T5	*TraesCS4A02G277600*	*TraesCS4B02G035500*	*TraesCS4D02G033500*		*TaCLC-4A1*	*TaCLC-4B1*	*TaCLC-4D1*	
TaCLC-T6	*TraesCS5A02G449500*	*TraesCS5B02G457100*	*TraesCS5D02G456000*		*TaCLC-5A1*	*TaCLC-5B1*	*TaCLC-5D1*	
TaCLC-T7	*TraesCS6A02G098500*	*TraesCS6B02G126400*	*TraesCS6D02G084300*		*TaCLC-6A1*	*TaCLC-6B1*	*TaCLC-6D1*	
TaCLC-T8	*TraesCS6A02G098600*	*TraesCS6B02G126800*	*TraesCS6D02G084000*		*TaCLC-6A2*	*TaCLC-6B2*	*TaCLC-6D2*	
TaCLC-T9	*TraesCS6A02G283600*	*TraesCS6B02G312100*	*TraesCS6D02G264100*		*TaCLC-6A3*	*TaCLC-6B3*	*TaCLC-6D3*	
TaCLC-T10	*TraesCS7A02G240700*	*TraesCS7B02G136300*	*TraesCS7D02G239700*		*TaCLC-7A1*	*TaCLC-7B1*	*TaCLC-7D1*	
TaSLAC-T1	*TraesCS1A02G127500*	*TraesCS1B02G147400*	*TraesCS1D02G126500*		*TaSLAC-1A1*	*TaSLAC-1B1*	*TaSLAC-1D1*	
TaSLAC-D1	*TraesCS1A02G423000*	*TraesCS1B02G455100*			*TaSLAC-1A2*	*TaSLAC-1B2*		
TaSLAC-T2	*TraesCS1A02G423900*	*TraesCS1B02G456000*	*TraesCS1D02G432500*		*TaSLAC-1A3*	*TaSLAC-1B3*	*TaSLAC-1D3*	
TaSLAC-D2	*TraesCS1A02G424400*			*TraesCSU02G204200*	*TaSLAC-1A4*			*TaSLAC-Un1*
TaSLAC-T3	*TraesCS1A02G424500*	*TraesCS1B02G456500*	*TraesCS1D02G433100*		*TaSLAC-1A5*	*TaSLAC-1B5*	*TaSLAC-1D5*	
TaSLAC-T4	*TraesCS2A02G398000*	*TraesCS2B02G416100*	*TraesCS2D02G395700*		*TaSLAC-2A1*	*TaSLAC-2B1*	*TaSLAC-2D1*	
TaSLAC-T5	*TraesCS3A02G028100*	*TraesCS3B02G018300*	*TraesCS3D02G017800*		*TaSLAC-3A1*	*TaSLAC-3B1*	*TaSLAC-3D1*	
TaSLAC-T6	*TraesCS3A02G151400*	*TraesCS3B02G178600*	*TraesCS3D02G159600*		*TaSLAC-3A2*	*TaSLAC-3B2*	*TaSLAC-3D2*	
TaSLAC-T7	*TraesCS3A02G167000*	*TraesCS3B02G199200*	*TraesCS3D02G174800*		*TaSLAC-3A3*	*TaSLAC-3B3*	*TaSLAC-3D3*	
TaSLAC-T8	*TraesCS3A02G225100*	*TraesCS3B02G254700*	*TraesCS3D02G228400*		*TaSLAC-3A4*	*TaSLAC-3B4*	*TaSLAC-3D4*	
TaSLAC-D3		*TraesCS1B02G456100*	*TraesCS1D02G432700*			*TaSLAC-1B6*	*TaSLAC-1D6*	
TaSLAC-S1		*TraesCS1B02G388600*				*TaSLAC-1B7*		
TaSLAC-T9		*TraesCS1B02G456200*	*TraesCS1D02G432900*	*TraesCSU02G001500*		*TaSLAC-1B8*	*TaSLAC-1D8*	*TaSLAC-Un2*
TaSLAC-T10		*TraesCS1B02G456400*	*TraesCS1D02G432800*	*TraesCSU02G001400*		*TaSLAC-1B9*	*TaSLAC-1D9*	*TaSLAC-Un3*
TaSLAC-T11		*TraesCS1B02G456300*	*TraesCS1D02G433200*	*TraesCSU02G001600*		*TaSLAC-1B10*	*TaSLAC-1D10*	*TaSLAC-Un4*

### Structure prediction of nitrate transporter proteins

Due to the unavailability of crystal structures, gene homology modelling was carried out to predict their three-dimensional (3D) structure. The sequences of *TaNRT1*, *TaCLC*, *TaSLAC1/TaSLAH* and *TaNRT2* genes were submitted to web-based server Phyre2^41^. Briefly, Phyre2 used PSI-BLAST to detect sequence homologues which was followed by Psi-pred and Diso-pred to predict secondary structure and disorder. Then Hidden Markov models (HMM) of sequences were generated based on homologues detected before. HMMs of query proteins were scanned against library of HMMs of proteins with experimentally solved structures to construct 3D models of query proteins. Transmembrane helix and topology prediction was carried by memsat-svm^41^.

### Expression analysis of nitrate transporter genes

The RNAseq data of *TaNPF*, *TaNRT2*, *TaCLC* and *TaSLAC1/TaSLAH* genes of various tissues (root, shoot/leaf, spike, grain) at three developmental stages (seedling, vegetative and reproductive) for Chinese spring and Azhurnaya (cv) was downloaded from the wheat expression database (www.wheat-expression.com). Expression levels were downloaded as log_2_(transcripts per million) (log_2_tpm) for different tissues at different time points. Several tissue-specific (root, shoot, leaf, grain) genes were identified based on expression patterns. For triad expression analysis, a method described by Ramírez-González et al.^42^ was used. Briefly, the expression data from spring wheat (CS) and Azhurnaya was downloaded from the wheat expression database as TPM for root, leave, shoot spike and grain. For analysis, the triads with expression below one tpm were excluded. Expression values were normalized, triads were assigned balanced, A/B/D suppressed or A/B/D dominant profiles. To elucidate the role of Nitrate transporter genes towards N starvation and N recovery, the gene expression data set^34–36^ from wheat omics 1.0 database (http://wheatomics.sdau.edu.cn/) was analysed. The dataset contained expression data in roots of 10-day old wheat plants (Chinese Spring) treated for N-starvation for 5 days and then subjected for N-recovery^34–36^.

### Development of validation panel to check the efficacy of the identified nitrate transporter genes

The nested synthetic hexaploid wheat (N-SHW) introgression library constituting a set of 352 breeding lines derived from four sub-populations (Pop1: 75 lines from PDW233/*Ae. tauschii* acc. pau 14,135 amphiploid //2*BWL4444; Pop2: 106 lines from PDW233/*Ae. tauschii* acc. pau 14,135 amphiploid //2*BWL3531; Pop3: 88 lines from PBW114/*Ae. tauschii* acc. pau 14,170 amphiploid //2*BWL4444; Pop4: 83 lines from PBW114/*Ae. tauschii* acc. pau 14,170 amphiploid //2*BWL3531) were developed^43^. These N-SHW library, six parents and two synthetic hexaploid wheats were assessed over 2 years in 2018 and 2019 at 3 nitrogen levels [i.e., zero N (0 kg ha^−1^), half N (60 kg ha^-1^) and full N (recommended, 120 kg ha^−1^]. The detailed phenotyping of the N-SHW introgression libraries for the nitrogen-use efficiency related traits was carried out across years and treatments^43^. High-density genotyping was performed using the 35 K Axiom® Wheat Breeder’s Array (Affymetrix UK Ltd., United Kingdom). The population structure of the 352 N-SHW lines was assessed on the basis of 9,474 SNPs distributed across all 21 wheat chromosomes. The most appropriate K explaining the population structure was K = 3 at MAF ≥ 5% **(**Supplementary Fig. 4A**)**. The kinship heatmap suggested a weak relatedness in the panel **(**Supplementary Fig. 4B**)**. The first three principal components (PCs) were most informative gradually decreasing **(**Supplementary Fig. 4C,D**)** until the tenth PC. The kinship and PCs were considered during the GWAS analysis to correct for population structure. The appropriate number of sub-populations was determined from the largest *delta K* value of 3 **(**Supplementary Fig. 4E**)**. The kinship and PCs were considered during the GWAS analysis to identify population structure. Significant marker-trait associations were identified using CMLM (compressed mixed linear model)/P3D (population parameters previously defined) in GAPIT (Genome Association and Prediction Integrated Tool) executed in R. Over 322 marker trait associations for NUE were compared to nitrate transporter genes.

## Results

### The wheat genome consists of 412 nitrate transporter genes belonging to four different families

**Figure 1:**
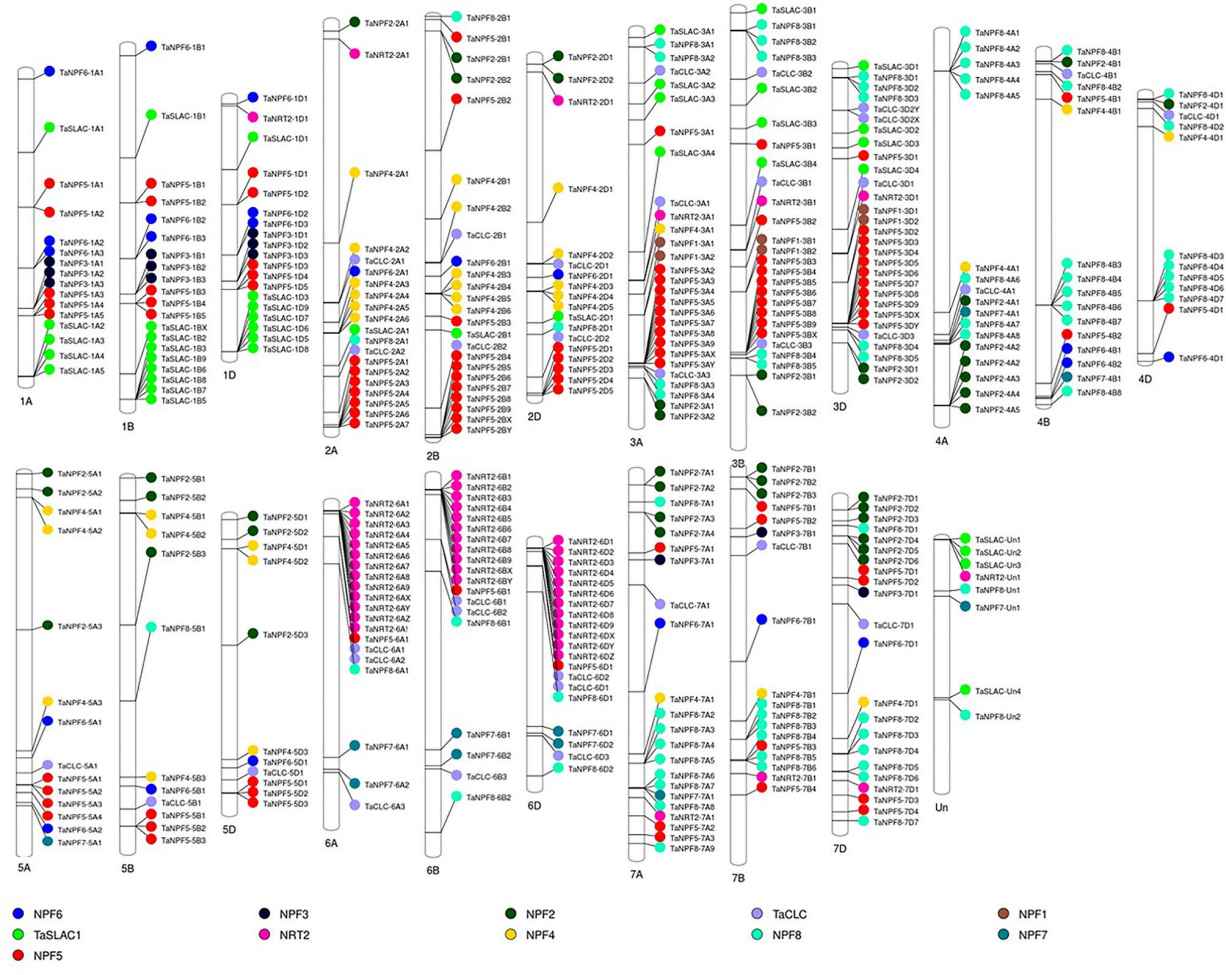
Genome wide distribution of *TaNPF*, *TaNRT2*, *TaCLC* and *TaSLAC1/TaSLAH* genes in hexaploid wheat. Figure was generated by web-based software tool-Phenogram from Ritchie Lab^44^ (http://visualization.ritchielab.org/phenograms/plot).

A total of 412 nitrate transporter sequences excluding splice variants were identified in IWGSC wheat genome assembly (RefSeq V2.0). The wheat genome consists of 292 *TaNPF* genes, 34 *TaCLC* genes, 40 *TaSLAC1/TaSLAH* genes and 46 *TaNRT2* genes. The *TaNPF* genes could be divided into eight subgroups (*TaNPF1* to *TaNPF8*) based on the presence of conserved domains (Table 1). *TaNPF5* subgroup was the largest group consisting of 97 genes followed by *TaNPF8* (70 genes), *TaNPF2* (41 genes), *TaNPF4* (33 genes), *TaNPF6* (22 genes), *TaNPF3* (12 genes) and *TaNPF7* (11 genes). The *NPF1* subgroup was the smallest one consisting of 6 genes present on homoeologous group chromosomes 3A, 3B and 3D. *TaNRT1/TaNPF* genes were present throughout the genome (Fig. 1). The location of genes across chromosomes varied according to the size of the subfamily. The genes belonging to larger subfamilies (e.g., *TaNPF5, TaNPF8, TaNPF2*) were predominantly located in tandem positions on the distal region of chromosomes. The genes belonging to smaller subfamilies (*TaNPF1, TaNPHF7, TaNPF3*) were located on proximal regions of chromosomes. The genes present near distal ends of chromosomes were found to be in the form of clusters in close vicinity to each other. The majority of *TaNRT2* genes were present in the clusters on the distal end of homoeologous chromosomes 6A, 6B and 6D. *TaCLC* genes were distributed across the wheat genome. *TaSLAC1/TaSLAH* genes were only distributed on homoeologous chromosomes 1A,1B, 1D, 2A, 2B, 2D, 3A, 3B and 3D. The predicted gene structures contained several intron regions (Supplementary Fig. 1a–c) for many genes in *TaNPF*, *TaCLC* and *TaSLAC1/TaSLAH* families. All the *TaNRT2* genes were intron less. The size of predicted genes ranged between 1 and 25 Kb. Several truncated and duplicated genes were also predicted.

### Phylogenetic relationships among nitrate transporter genes

**Figure 2:**
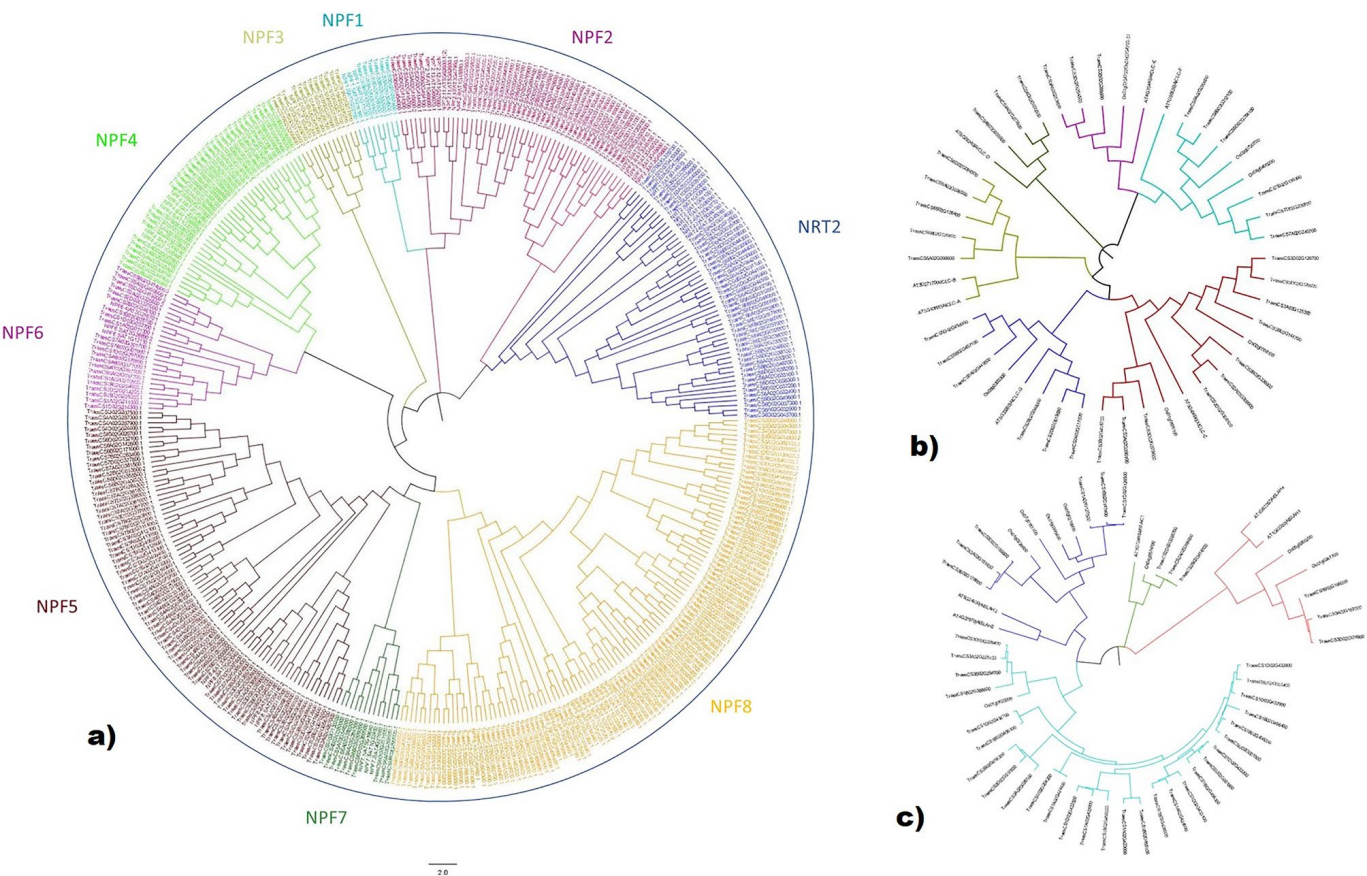
Phylogenetic tree depicting relationship between (**a**) *TaNPF* and *TaNRT2* genes in hexaploid wheat and *Arabidopsis thaliana* (**b**) *TaCLC* genes in wheat, rice and *Arabidopsis thaliana* (**c**) *TaSLAC1/SLAH* genes in hexaploid wheat, rice and *Arabidopsis thaliana.* Phylogenetic analysis was performed by MEGA X software^45^ and the results were edited and visualized by FIGTREE software v1.4.4. (http://tree.bio.ed.ac.uk/software/figtree/) to generate final images.

The maximum likelihood phylogenetic tree of all the nitrate transporter genes predicted that wheat contains all the major subfamilies present in *Arabidopsis* and rice (*Oryza sativa*) (Fig. 2a). The *TaNRT1/TaNPF* and *TaNRT2* genes could be classified into five subclades. The subclades in the phylogenetic tree followed species phylogeny with *Arabidopsis* genes displaying sister group relationship with wheat genes. Based on the phylogenetic relationship, *TaNRT1/TaNPF* genes fitted well into eight subfamilies (*TaNPF1* to *TaNPF8*) following the *Arabidopsis* model. The topology of larger subclades (*TaNPF5*, *TaNPF8*, *TaNPF2*) was more complex than smaller subclades as they were more expanded in wheat than *Arabidopsis* and rice (Fig. 2a, Supplementary Fig. 2). *TaNRT2* genes were present as a separate subclade and were closely related to the *TaNPF2* subfamily. The phylogenetic analysis of *TaCLC* and *TaSLAC1/TaSLAH* genes was carried out separately. The results showed *TaCLC* genes could be classified into 6 groups according to phylogenetic relation with *Arabidopsis* and rice genes (Fig. 2b). *TaSLAC1/TaSLAH* genes were divided into 4 subclades. The largest subclade in *TaSLAC1/TaSLAH* genes showed close relationship with rice *SLAC1/SLAH* genes but not with *Arabidopsis* genes (Fig. 2c).

### Homoeologs retention and gene duplication in nitrate transporter genes

**Figure 3:**
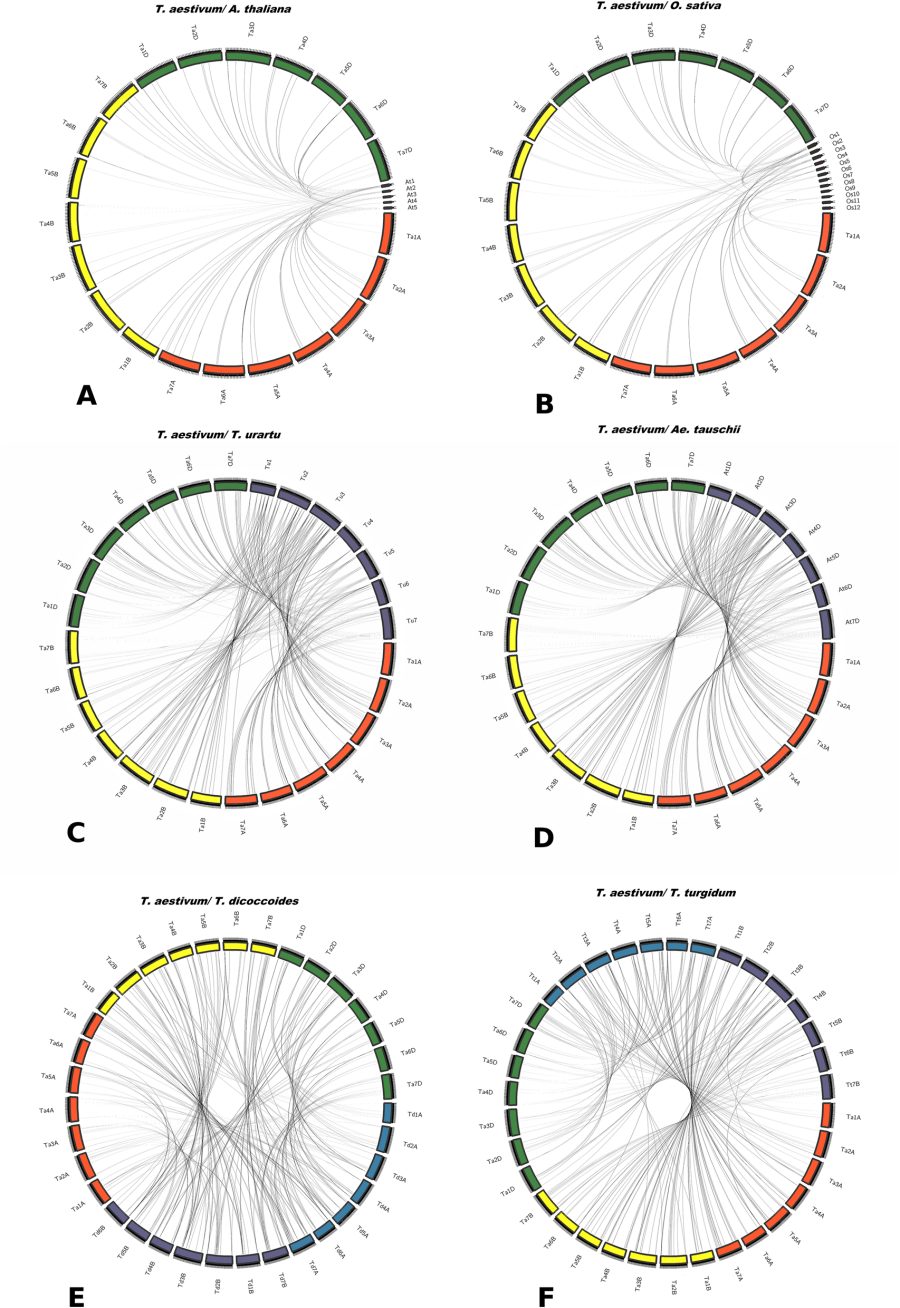
Synteny relationships of wheat nitrate transporter genes orthologous with (**A**) *A. thaliana,* (**B**) *O. sativa,* (**C**) *T. urartu*, (**D**) *Ae. tauschii,* (**E**) *T. dicoccoides* and, (**F**) *T. turgidum.* Circos plots were generated by web-based application- shinyCircos (https://venyao.xyz/shinycircos/)^46^.

The number of nitrate transporter genes in each family were significantly higher than those in *Arabidopsis* and rice (Table 1, Supplementary Table 1). The comparison with *T. dicoccoides* (AABB), *T. turgidum* (AABB), *T. urartu* (AA) and *Ae. tauschii* (DD) suggested that most of the homoeologs in hexaploid wheat were retained during evolution (Fig. 3, Supplementary Table 1). There was also evidence of gene duplications in tetraploids and hexaploid wheat, reflected in gene number and phylogenetic data (Fig. 2, Supplementary Fig. 1a–c). Most duplicated genes were present in subfamilies with a larger number of genes (*TaNPF5*, *TaNPF8*, *TaNPF2* and *TaNRT2*). Nitrate transporters could be grouped into 13 triads, 26 diads, 2 tetrads and 48 singleton genes based on phylogeny (Table 3). Out of a total of 292 *TaNPF* genes, about 74% of *TaNPF* genes could be grouped into 72 triads of homoeologous genes (A, B, D) based on phylogenetic relationships. Similarly, 71% of *TaNRT2* genes, 97% of *TaCLC* genes and 80% of *TaSLAC1/TaSLAH* genes could be grouped into homoeologous triads.

**Table 3:** Number of triads, tetrads, diads and singletons detected in nitrate transporter families in hexaploid wheat genome.

Family/ Subfamily	No. of Triads	No. of diads			No. of Tetrads			Singletons			
	A:B:D = (1:1:1)	A:B:D = (1:1:0)	A:B:D = (1:0:1)	A:B:D = (0:1:1)	A:B:D = (1:1:2)	A:B:D = (1:2:1)	A:B:D = (2:1:1)	A:B:D = (1:0:0)	A:B:D = (0:0:1)	A:B:D = (0:1:0)	Un
*TaNPF1*	2	0	0	0	0	0	0	0	0	0	1
*TaNPF2*	9	0	2	2	0	0	0	5	1	0	0
*TaNPF3*	4	0	0	0	0	0	0	0	0	0	0
*TaNPF4*	10	1	0	0	0	0	0	1	0	0	0
*TaNPF5*	22	1	1	6				2	12	1	0
*TaNPF6*	6	0	0	0	0	0	0	1	2	1	0
*TaNPF7*	2	0	0	0	0	0	0	3	1	1	1
*TaNPF8*	17	1	1	3				4	2	1	2
*TaNRT2*	10	2	3	0	1	0	0	0	0	0	1
*TaCLC*	10	0	0	0	1	0	0	0	0	0	0
*TaSLAC1/TaSLAH*	11	1	1	1	0	0	0	0	0	1	4
Total	103	6	8	12	2	0	0	16	18	5	9

### Nitrate transporter proteins contain multiple transmembrane helices

**Figure 4:**
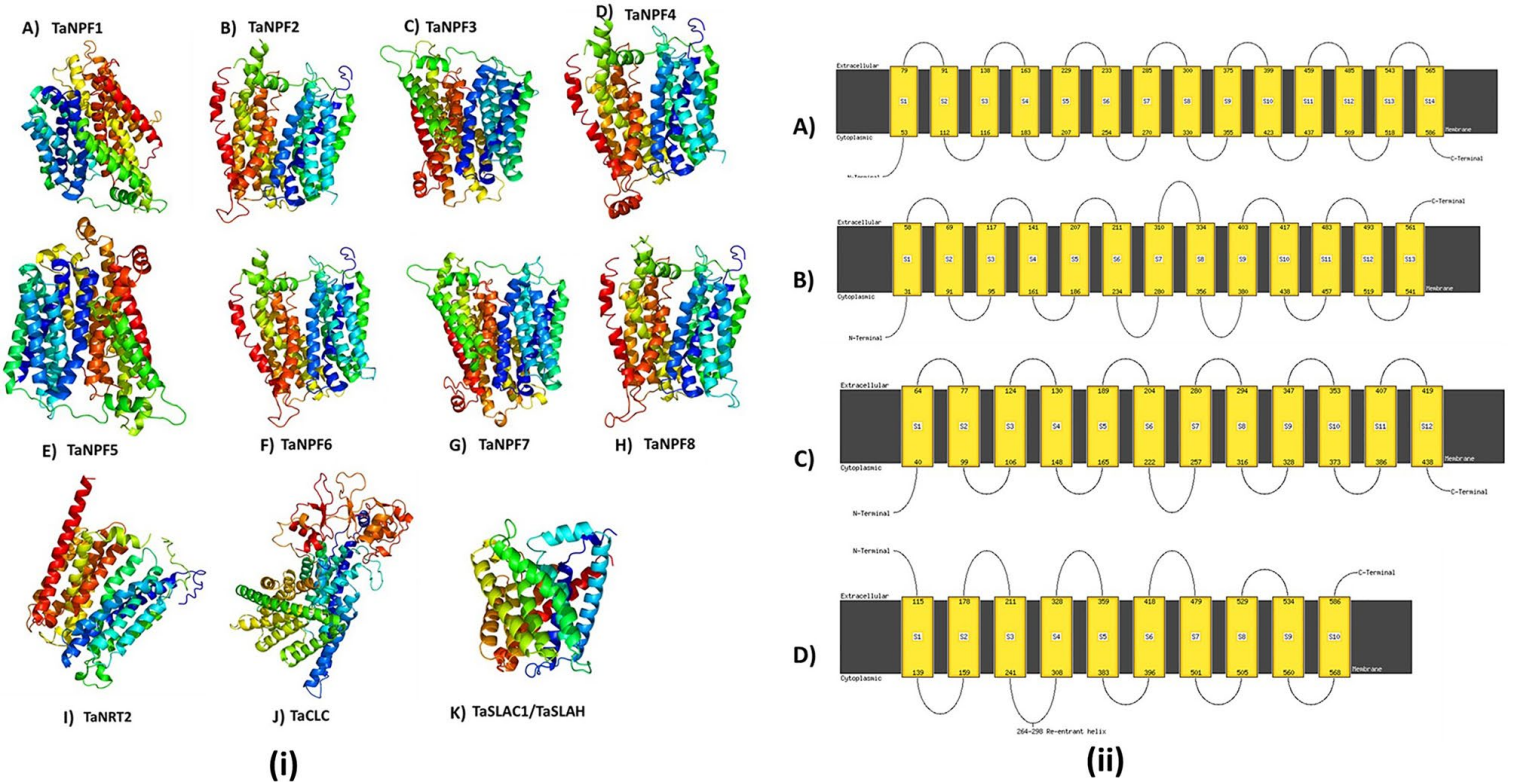
Protein structure prediction: (i) representative structures of *TaNPF* genes (**A**–**H**), *TaNRT2* genes (**I**) *TaCLC* genes (**J**) and *TaSLAC1/TaSLAH* genes (**K**). (ii) Representative TMs structures of nitrate transporters containing (**A**) 14 TMs, (**B**) 13 TMs (**C**) 12 TMs and (**D**) CLC proteins containing 10 TMs and a re-entrant helix. Figures were developed by homology-based modelling by Phyre2 server^41^.

To study the structural features of nitrate transporters, we predicted the 3D structures of all 412 protein sequences. All nitrogen transporters were predicted to be transmembrane proteins containing multiple transmembrane segments (Fig. 4i). The majority of proteins comprised of 12–14 transmembrane helices (TMs) with some variation. The basic structure of *TaNRT/TaNPF* proteins included N and C terminal segments followed by multiple transmembrane helices (TMs). The transmembrane helices were connected by alternating cytoplasmic and extracellular loop segments (Fig. 4ii). In *TaNRT1/TaNPF* family, approximately 67% of the proteins contained 14 TMs, 21% contained 13 TMs, 7% of proteins contained 12 TMs while 4% of proteins contained less than 12 TMs (Supplementary Table 2). Subfamily wise studies showed *TaNPF1* proteins contained only 13 TMs and *TaNPF7* contained only 14 TMs. In rest of subfamilies (*TaNPF2-6, TaNPF8*) majority of proteins contained 14 TMs but variation existed. Proteins with even number of TMs had both C and N terminals in cytoplasmic side of membrane. Proteins with odd number of TMs had one end in cytoplasmic side and other in extracellular side (Fig. 4ii). All *TaNRT2* family members contained only 12 TMs (Supplementary Table 2) (Fig. 4ii). Both C and N terminals of *TaNRT2* proteins were present in cytoplasmic side of the membrane. Both *TaCLC* and *TaSLAC1/SLAH* proteins contained 10 TMs with both N and C terminals in cytoplasmic side of membrane. *TaCLC* genes were characterized by presence of a 30–40 amino acids long re-entrant helix in cytoplasmic side (Fig. 4 ii) which was not observed in the proteins of other nitrate transporter gene families.

### Expression patterns of nitrate transporter genes in development stages of wheat

**Figure 5:**
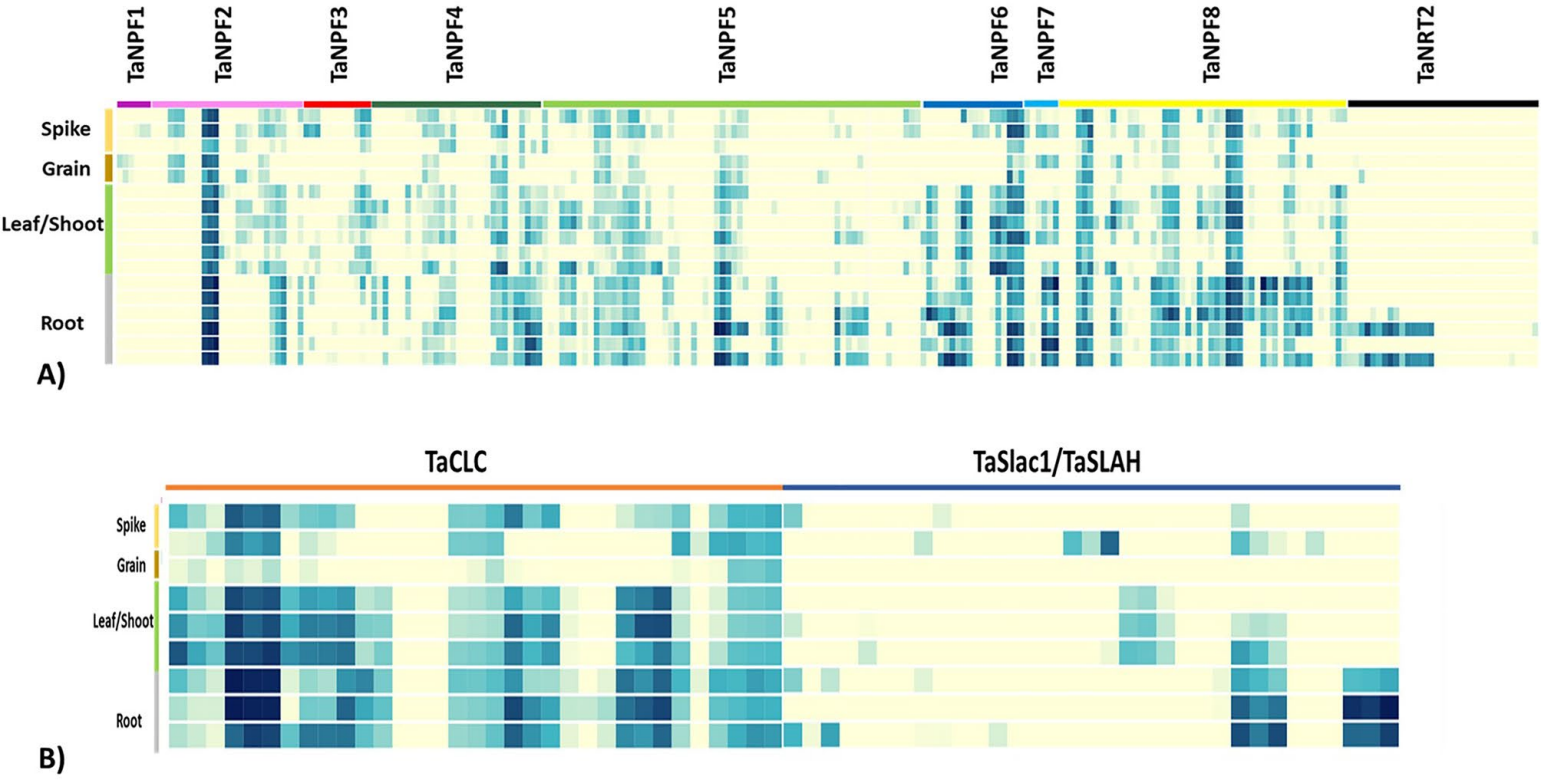
Expression patterns of nitrate transporter gene triads in wheat (**a**) Tissue and development stage specific expression profiles of *TaNPF* and *TaNRT2* genes (**b**) Tissue and development stage specific expression profiles of *TaCLC* and *TaSLAC1/SLAH* genes. The heat maps were generated by heatmap tool from wheat expression database^42^ (http://wheat-expression.com/).

To elucidate the expression patterns of nitrate transporter genes, we studied and compared the expression data of Chinese spring and Azhurnaya for different developmental stages. Approximately 77% of *TaNPF* genes, 30% of *TaNRT2*, 85% of *TaCLC* genes and 36% of *TaSLAC1/TaSLAH* genes were expressed at least at one developmental stage in wheat with a wide expression range of 1–103 tpm (Supplementary Table 3, Supplementary Fig. 3). The remaining genes showed very low or no expression (tpm < 1). Overall, we identified 20 triads in which 48 genes were showing tissue specific expression, out of which 8 triads were root specific, 5 triads were leaf/shoot specific and 7 triads were showing grain/ spike specific expression (Supplementary table 4). Tissue and developmental stage-specific expression were observed in *TaNPF1* genes, which were only expressed in spike and grain at the reproductive stage (Fig. 5A). Similarly, *TaNRT2* genes were predominantly expressed in roots in both vegetative and reproductive stages (Fig. 5A). *TaSLAC1/TaSLAH* genes were predominately expressed in roots and leaves with some genes showing expression in spikes also (Fig. 5B). *TaCLC* genes showed mostly ubiquitous expression (Fig. 5B). For the rest of the subfamilies, the genes within one subfamily differed considerably in their expression patterns. In *TaNPF2* genes, spike/grain specific (3 genes), leaf, spike and grain specific (5 genes) and ubiquitous expression (6 genes) were observed (Fig. 5A). *TaNPF3* genes showed spike/grain, leaf specific expression, *TaNPF4* genes showed leaf/root-specific (4 genes) and ubiquitous expression (10 genes) (Fig. 5A). *TaNPF5* and *TaNPF8* genes mostly showed ubiquitous expression though the root-specific expression was observed in a few genes (Fig. 5A). *TaNPF6* showed ubiquitous (6 genes), leaf and root-specific (6 genes), spike specific (3 genes) and root-specific expression (Fig. 5A). *TaNPF7* showed ubiquitous expression in three genes, grain specific expression in two genes and root-specific expression in one gene (Fig. 5A).

**Figure 6:**
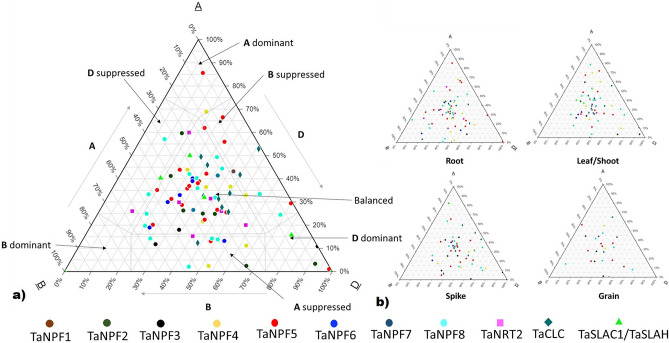
Triad expression of nitrate transporters in wheat (**A**) Overall triad expression of all nitrate transporter genes (**B**) Tissue specific triad expression of nitrate transporter genes. Normalized expression values were used to generate ternary plots using online web-based tool (https://www.ternaryplot.com/).

To find out up to what extent homoeologs differ in the expression patterns, triad expression analysis was performed. Most of the triads showed balanced expression ranging from 55.6 to 65.2% in all the tissues (Fig. 6A). In roots, a total of 54 triads were showing expression out of total 83 triads. Out of which 55.6% showed balanced expression, 18.5% showed A suppressed, 11.1% showed D suppressed, 9.3% showed B suppressed expression. Three triads showed A, B and D dominant expression (1 each) (Fig. 6B). In leaf/shoot out of 51 triads, 64.7% showed balanced expression, 9.8% showed A suppressed and B suppressed each, 3.9% triads showed D suppressed expression. 5.8% triads showed A and D dominant expression each while no B dominant expression was observed (Fig. 6B). In spikes, 61.9% triads out of 42 triads showed balanced expression. Only D dominant expression was observed in 9.5% of triads while A suppressed, B suppressed, and D suppressed expressions were in about 16.7, 7.1% 4.7% triads (Fig. 6B). Only 23 triads were expressing in grains at the reproductive stage, out of which 65.2% showed balanced expression, 8.7% triads showed A, B, and D suppressed each and 4.3% triads showed B and D dominant expression (Fig. 6B).

### Nitrate transporter genes are located in close proximity to the NUE associated SNPs

In a parallel study in our laboratory, the nested synthetic wheat introgression (N-SHW) libraries capturing novel genetic variation from wild wheat for the nitrogen use efficiency related traits were developed and genotyped using a high-density SNP array^43^. These libraries were phenotypically assessed for the root traits and agronomic performance under three nitrogen input conditions (N: 0 kg ha^−1^; N: 60 kg ha^−1^ and N:120 kg ha^−1^) in the field over two years in 2018 and 2019. Genome-wide association mapping was used to identify marker-trait associations for the root and agronomic traits to identify the marker-trait associations for traits improving nitrogen use efficiency in wheat (Supplementary Table 5). We compared 322 marker trait associations for NUE identified in this study^43^ to nitrate transporter genes identified during genome wide analysis. We identified 67 SNPs, which were in close proximity to nitrate transporter genes in the wheat genome. A total of 93 nitrate transporter genes could be located near NUE linked SNPs, out of which, 63 genes belonged to *TaNPF* family, 15 genes belonged to *TaNRT2* family, 11 genes belonged to *TaCLC* and 4 genes belonged to TaSLAC1/TaSLAH family (Table 4, Supplementary Fig. 5).

**Table 4:** Proximity of nitrogen use efficiency (NUE) linked SNPs^43^ to nitrate transporters detected in present study.

SNP related to NUE	Chromosome	SNP Position	Nearby nitrate transporters	Nitrate transporter Position	Distance (in Mb)
AX94950355	1A	12918698	*TaNPF6-1A1*	14519757	1.601059
AX94815202	1A	14468156	*TaNPF6-1A1*	14519757	0.051601
AX94665912	1B	624080881	*TaSLAC-1B10*	622365197	1.715684
AX94923560	2A	729858424	*TaCLC-2A2*	740847366	10.988942
AX94906008	2A	737049474	*TaCLC-2A2*	740847366	3.797892
AX95162328	2A	745066946	*TaCLC-2A2*	740847366	4.21958
AX94601746	2B	745715147	*TaCLC-2B2*	742813858	2.901289
AX95203088	2B	748700718	*TaCLC-2B2*	742813858	5.88686
AX95190948	2B	752830609	*TaCLC-2B2*	742813858	10.016751
AX95189671	2D	394797805	*TaNPF4-2D2, TaCLC-2D1*	394118961 395130092	0.332287, 0.678844
AX94829391	2D	601212191	*TaCLC-2D2*	608915455	7.703264
AX95142803	2D	601600533	*TaCLC-2D2*	608915455	7.314922
AX94799671	2D	608756380	*TaCLC-2D2*	608915455	0.159075
AX95142189	2D	609577225	*TaCLC-2D2*	608915455	0.66177
AX94786006	2D	610277424	*TaCLC-2D2*	608915455	1.361969
AX95148777	2D	641963392	*TaNPF5-2D1-TaNPF5-2D5*	639677529–643761743	1.798351–2.285863
AX95238274	3A	429463868	*TaSLAC-3A4*	421719078	7.74479
AX94593608	3A	671144035	*TaNPF2-3A1, TaNPF2-3A2*	660436466 660507764	10.636271, 10.707569
AX95237615	3B	6378879	*TaSLAC-3B1*	7598907	1.220028
AX95259763	3B	229302401	*TaSLAC-3B3*	227663976	1.638425
AX95136655	3B	235865416	*TaSLAC-3B3*	227663976	8.20144
AX94723497	3B	236511642	*TaSLAC-3B3, TaNPF3B1*	227663976	8.847666
AX94561045	3B	642481079	*TaNPF5-3B3–TaNPF5-3B10, TaCLC-3B3*	651425224–655435367	8.944145–12.954288
AX94539428	3B	657947249	*TaCLC-3B3, TaNPF5-3B3–TaNPF5-3B10, TaNPF-3B4, TaNPF-3B5*	651425224–662795946	2.511882–6.522025
AX94386613	3B	658604225	*TaCLC-3B3, TaNPF5-3B3–TaNPF5-3B10, TaNPF-3B4, TaNPF-3B5*	651425224–662795946	3.168858–7.179001
AX94418180	3B	659275308	*TaCLC-3B3, TaNPF5-3B3–TaNPF5-3B10, TaNPF-3B4, TaNPF-3B5*	651425224–662795946	3.839941–7.850084
AX94429243	3B	659787974	*TaCLC-3B3, TaNPF5-3B3–TaNPF5-3B10, TaNPF-3B4, TaNPF-3B5*	651425224–662795946	4.352607–8.36275
AX94910184	3D	352948426	*TaCLC-3D1, TaNRT2-3D1*	355885478 356623041	2.937052, 3.674615
AX94514369	4A	544201715	*TaNPF4-4A1*	533257983	10.943732
AX94926692	4A	544202284	*TaNPF4-4A1*	533257983	10.944301
AX94766675	4A	575009572	*TaNPF8-4A6*	575006132	0.00344
AX94400142	4A	581754986	*TaCLC-4A1, TaNPF2-4A1, TaNPF7-4A1, TaNPF8-4A7, TaNPF8-4A8*	585431883–593113134	3.676897–11.358148
AX94414780	4B	25929732	*TaCLC-4B1, TaNPF2-4B1, TaNPF4B1*	20278828–25842359	5.650904
AX94478236	4B	28716503	*TaCLC-4B1, TaNPF2-4B1, TaNPF4B1*	20278828–25842359	2.874144–8.437675
AX94997694	4B	34789538	*TaCLC-4B1, TaNPF2-4B1, TaNPF4B1*	20278828–25842359	8.947179–14.51071
AX94517352	4D	21886662	*TaCLC-4D1, TaNPF2-4D1, TaNPF8-4D1*	10764927–15356868	6.529794–11.121735
AX94586364	4D	22947854	*TaCLC-4D1, TaNPF2-4D1, TaNPF8-4D1*	10764927–15356868	7.590986–12.182927
AX94914919	4D	28974006	*TaNPF8-4D2*	28481269	0.492737
AX94738199	5D	10899555	*TaNPF2-5D1*	6820550	4.079005
AX95110067	5D	467774783	*TaNPF4-5D3*	464415850	3.358933
AX95002541	5D	468689841	*TaNPF4-5D3*	464415850	4.273991
AX95132327	5D	472234562	*TaNPF4-5D3*	464415850	7.818712
AX94631745	5D	528728651	*TaNPF5-5D1—TaNPF5-5D3*	528294425*–*528587054	0.141597–0.43423
AX94803288	6A	14353974	*TaNRT2-6A1- TaNRT2-6A13*	15727844–16408185	1.37387–2.054211
AX95017906	6A	23433182	*TaNRT2-6A14*	21634811	1.798371
AX94983341	6A	28412753	*TaNRT2-6A14*	21634811	6.777942
AX95210745	6A	29967076	*TaNRT2-6A14*	21634811	8.332265
AX94510892	6A	112585030	*TaNPF8-6A1*	117412062	4.827032
AX94534539	6A	497462168	*TaNPF7-6A1*	486547388	10.91478
AX94573487	6D	27978202	*TaNPF5-6D1*	22172543	5.805659
AX94415776	6D	28700804	*TaNPF5-6D1*	22172543	6.528261
AX94978974	6D	29876083	*TaNPF5-6D1*	22172543	7.70354
AX94737868	6D	29876631	*TaNPF5-6D1*	22172543	7.704088
AX95250225	6D	29928065	*TaNPF5-6D1*	22172543	7.755522
AX94461279	6D	451183032	*TaNPF8-6D2*	449226044	1.956988
AX94665619	7A	222939896	*TaCLC-7A1*	216343576	6.59632
AX94566038	7A	683488235	*TaNPF5-7A3*	692626752	9.138517
AX95178548	7B	112337703	*TaNPF5-7B2*	116046396	3.708693
AX94532247	7B	524619772	*TaNPF8-7B1- TaNPF8-7B3*	517623485–518338133	6.281639–6.996287
AX94424632	7B	562740463	*TaNPF8-7B4–TaNPF8-7B6*	556686657- 558639959	4.100504–6.053806
AX94880654	7B	592345313	*TaNRT2-7B1*	583923053	8.42226
AX94553632	7B	633318727	*TaNPF5-7B4*	624468356	8.850371
AX94781629	7D	206695834	*TaCLC-7D1*	204246408	2.449426
AX94678472	7D	487753483	*TaNPF8-7D2*–*TaNPF8-7D4*	489153269–489673028	1.39978- 1.91954
AX95080011	7D	592191388	*TaNPF5-7D4*	600836846	8.645458

### Response of nitrate transporter genes during N-starvation and N-recovery

**Figure 7:**
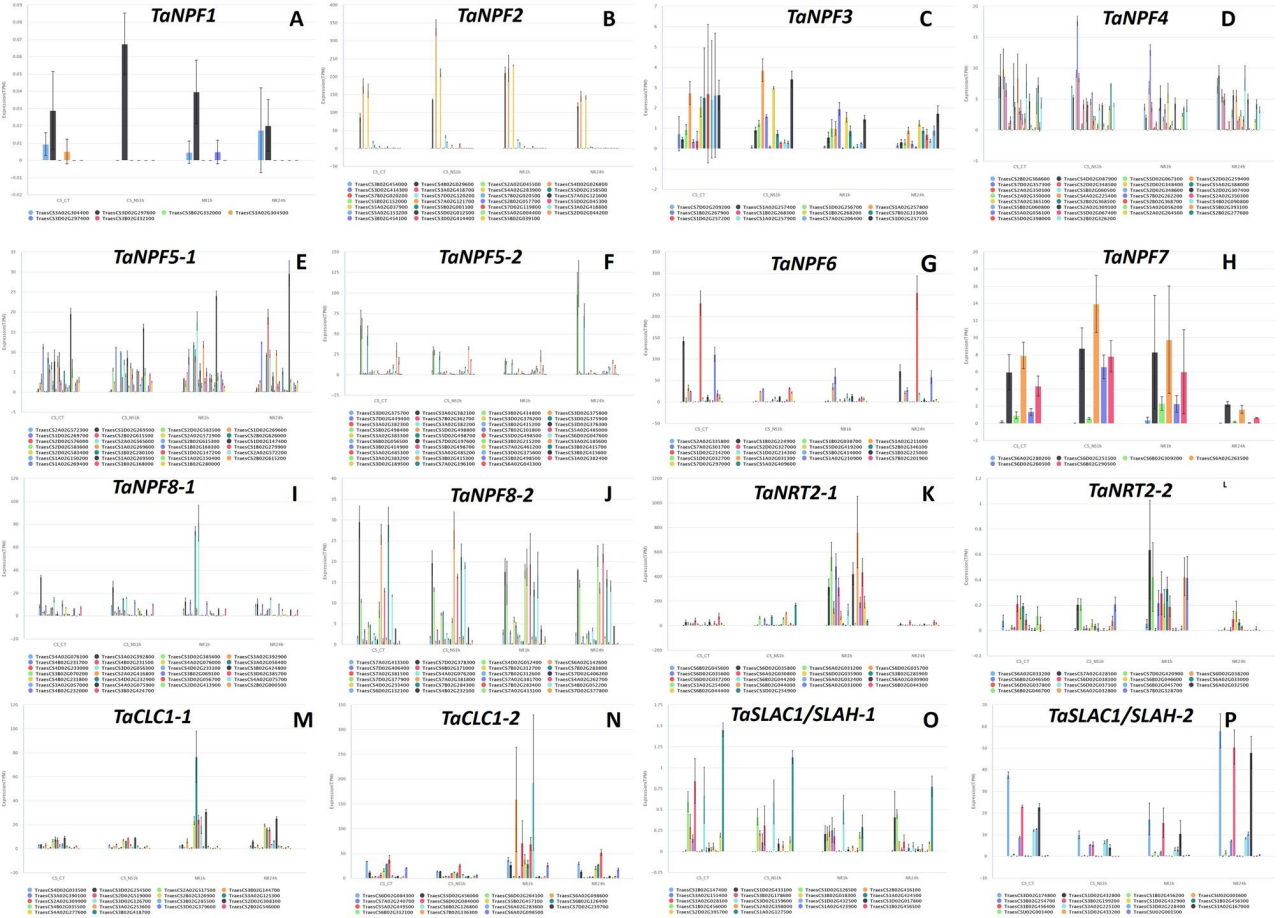
Expression profiles of nitrate transporter genes in response to Nitrogen starvation and Nitrogen recovery. The graphs were generated by GeneExpression tool from WheatOmics 1.0 database^47,48^.

The response of all N transporter genes towards N starvation and N recovery was analysed from WheatOmics database^34–36,47,48^. The results suggested that the expression of N transporter genes towards N starvation and N recovery was variable. We specifically identified the genes whose expression patterns changed significantly in response to N starvation or N recovery. The expression values of *TaNPF1* and *TaNPF3 genes* were not significant (Fig. 7A,C). Three genes in *TaNPF2* showed increased expression in N starvation and their expression values returned to normal during N recovery (Fig. 7B). The expression values of most of *TaNPF5* genes were slightly reduced during N starvation and increased significantly during N recovery (Fig. 7E,F). *TaNPF6* genes expression reduced during both N starvation and N recovery (1 h) but their expression returned to normal 24 h after recovery (Fig. 7G). The expression of most of *TaNPF7* genes was upregulated during N starvation and N recovery (1 h) and downregulated after 24 h of N recovery (Fig. 7H). The expression of *TaNPF4* and *TaNPF8* genes was variable (Fig. 7D,I,J). The expression of most of *TaNRT2* and *TaCLC* genes was upregulated during N recovery (1 h) phase (Fig. 7K,L,M,N). The expression values of some *TaSLAC1/TaSLAH* genes were reduced in response to N starvation and increased during N recovery (24 h) (Fig. 7O,P). Specifically looking into the expression pattern of 93 genes in close proximity of NUE associated SNPs, we could identify 32 genes whose expression pattern changed in response to N starvation and N recovery (Supplementary Fig. 6, Supplementary Table 6). These genes can serve as candidate genes and may be further utilized in genomics-assisted breeding programs targeting improved nitrogen-use efficiency in wheat.

## Discussion

The main aim of this study was to identify and analyse nitrate transporters belonging to all the four families and study their dynamics in wheat. The number of nitrate transporter genes detected in wheat was higher as compared to other plant species. This could be explained by a large genome (~ 18 Gb) and hexaploid nature of wheat. Presence of three homoeologous sub-genomes in wheat could allow multiple copies of nitrate transporters resulting in higher number of transporter genes. When comparing with diploid progenitors (*Ae. tauschii* and *T. urartu*) and tetraploid wheats (*T. dicoccoides* and *T. turgidum*) the number of genes in each subfamily were approximately proportional (Table 1). The genes were distributed randomly in the genome except for *TaNRT2* genes which were predominantly present on group 6 homoeologous chromosome. Many genes were present in form of clusters and showed high percentage of similarity indicating gene-duplication events. There were genes with deleted segments present in the genome. The phylogenetic relationships with orthologues in other plants could be used to classify the genes in subfamilies. All the major subclades were conserved in wheat in comparison to other plant species indicating biological importance of the subfamilies. Based on phylogeny the genes could be grouped in homoeologous triads. Almost 73% of the genes could be assigned to 1:1:1 homoeologous groups which is very much above the average homoeologous retention rate (35.8%) in wheat (IWGSC 2018). Many genes were also grouped into tetrads and diads based on homology indicating gene duplication and deletion events in the genome. The overall results revealed that wheat nitrogen transporter families are much more complex than in other plant species. This complexity arises mostly due to presence of three sub-genomes (A B D) and gene duplication and deletion events.

The complexity of wheat genome also affects the expression patterns of genes. Due to presence of multiple sets of homoeologs on A, B and D genomes the buffering effects are observed in expression of genes. To study up to what extent these interactions affect the expression of nitrate transporters, triad expression analysis was performed. More than 55% of genes showed balanced expression in all the tissues which is comparable to genome-wide assessment of all transcripts in wheat^42^. The expression profiles of the genes identified in this study were in accordance to the previous studies in other plants. The expression patterns of nitrate transporter genes were similar to expression patterns of close orthologs in rice and *Arabidopsis* indicating the conservation of gene functions. *CLC* genes in previous studies in *Arabidopsis* showed ubiquitous expression which was observed in this study for wheat as well^27,28^. Several tissue specific nitrate transporter genes were identified which can be targeted for gene manipulation for wheat improvement. Several *TaNRT2* and *TaSLAC1/TaSLAH* genes showed root specific expression suggesting their role in root nitrate uptake. Root specific expression of *NRT2* and *TaSLAC1/TaSLAH* genes has already been reported in rice and *Arabidopsis*^29,49^. *TaNPF1* genes and some *TaSLAC1/SLAH* genes showed grain and spike specific expression suggesting their role in nitrate transfer in developing seeds.

Structure plays a very important role in the function of transporter proteins. X-ray crystallographic structures of eukaryotic nitrate transporters have been elucidated^50^. All the nitrate transporter families belong to a much larger major facilitator superfamily (MFS) according to transporter classification database^51^. All the nitrate transporter proteins were predicted to have a typical MFS protein structure with multiple TMs. To the best of our knowledge our study is the first one to report homology-based models of nitrate transporter proteins belonging to all four families in wheat. The number of transmembrane segments play very important role in the optimal functioning MFS transporter proteins^52^. For an MFS transporter protein to have optimal transport properties pseudosymmetry is important which is provided by even number of TMs^50^. According to previous studies most of MFS proteins required 12 TMs to have optimal function^53^. In our study we predicted nitrate transporter families having variation in the number of TMs. *TaNPF* family being the largest of all showed most variation in the number of TMs with number ranging from 12 to 14. Several proteins with odd number of TMs were also observed. For example, all the members of *TaNPF1* subfamily contain 13 TMs. All *TaNRT2* proteins were highly conserved and contained 12 TMs. Most of the *TaCLC* and *TaSLAC1/TaSLAH* genes contained only 10 TMs. The variation in number of TMs between and within subfamilies and presence of odd number of TMs could not be corelated with expression data suggesting that a much more flexible criteria exists for the function of nitrate transporter proteins. The structural information presented in this study offer foundation for future work to identify molecular mechanisms responsible for functioning of nitrate transporters in wheat.

Previously in many studies overexpression of nitrate transporter genes has been linked to improved nitrogen use efficiency and yield in many plants^54–57^ and^58^. Overexpression of *OsNRT2.1*, *OsNRT2.3b*, *OsNPF6.3* in rice and *ZmNRT1.1A* in maize has resulted in increased grain yield^25,34–36,57,57,59^. In wheat *TaNRT2.1* is reported to be involved in post-flowering N uptake^32^ and is an important gene for improvement of nitrogen use efficiency. The *CLC* genes have been reported to be involved in nitrate accumulation in plants^26^ and many *CLC* genes have been reported to have role in stress responses. *SLAC1* is a key player in regulation of stomatal closure. *SLAH* genes are involved in root nitrate and chloride acquisition and translocation to shoot. *SLAC1/SLAH* genes have also been reported to have important role in drought responses^49^. The genome wide analysis of *TaCLC* and *TaSLAC1/TaSLAH* genes in this study is the first reported study of these genes in wheat to the best of our knowledge. Nitrate transporters identified in this study can be promising candidates for gene manipulation to enhance productivity and nitrogen use efficiency in wheat. The identification of nitrate transporter genes in the close proximity to the marker-traits associations indicated the robustness of genome wide association mapping studies and the reliability of the reported transporter genes. The identified nitrate transporters could deepen the understanding of genetic and molecular mechanism behind improving nitrogen-use efficiency in wheat crop. The nutrient efficient improved breeding lines/accessions possessing identified potential nitrate transporters in the present study may have an effective and strong coordinated signal transduction network involving nitrate transceptor, nitrate response regulator and the master response regulator.

 The *in-silico* mining of nitrate transporter genes along with their detailed structure, phylogenetic and expression studies reported a total of 412 nitrate transporter genes including 20 root specific, 11 leaf/shoot specific and 17 grain/spike specific putative candidate genes. The identification of nitrate transporter genes in the close proximity to the previously identified 67 marker-traits associations associated with the nitrogen use efficiency related traits in nested synthetic hexaploid wheat introgression library^43^ indicated the robustness of the reported transporter genes. The detailed crosstalk between the genome and proteome and the validation of identified putative candidate genes through expression and gene editing studies may lay down the foundation to improve nitrogen use efficiency of cereal crops. The existing genetic variability for 48 tissue specific genes and 93 genes in close proximity to NUE associated SNPs identified in the present study in different wild and cultivated wheat accessions/varieties may be further utilized in genomics-assisted breeding programs targeting improved nitrogen-use efficiency in wheat. A total of 32 genes out of these 93 genes show significant changes in expression patterns in response to N starvation and/ or N recovery suggesting their involvement in N uptake and assimilation. These genes can serve as initial candidates for targeting N use efficiency in wheat. The identification of improved breeding lines or the wild accessions possessing the potential nitrate transporters may serve as novel donors to be used in genomics-assisted introgression program developing nitrogen-efficient wheat varieties. The identified nitrate transporters may have potential for efficient nitrogen uptake and its transport from source to sink.

Once validated, the candidate genes may further be deployed in genomics-assisted breeding program to develop nutrient efficient wheat varieties. The present study provides important information on potential nitrate transporters that may lay foundation to develop a new breeding strategy for the sustainable agricultural development of cereal crops with less input—more output and the environmental protection. The identified nitrate transports may be of great significance both in the theory and in the genomics-assisted breeding application^39–26^.

## Supplementary Information

Supplementary Information 1.

Supplementary Information 2.

Supplementary Information 3.

## Data Availability

All data used in this research are included in this published article and its supplementary information files.
