# Supplementary material for: Genome-wide identification and in silico analysis of NPF, NRT2, CLC and SLAC1/SLAH nitrate transporters in hexaploid wheat (Triticum aestivum)
Source: Sci Rep. 2022 Jul 3;12:11227. doi: 10.1038/s41598-022-15202-w (PMC9250930; doi:10.1038/s41598-022-15202-w)
Supplement: Supplementary file 1 — Supplementary Information 1. [file 41598_2022_15202_MOESM1_ESM.docx]

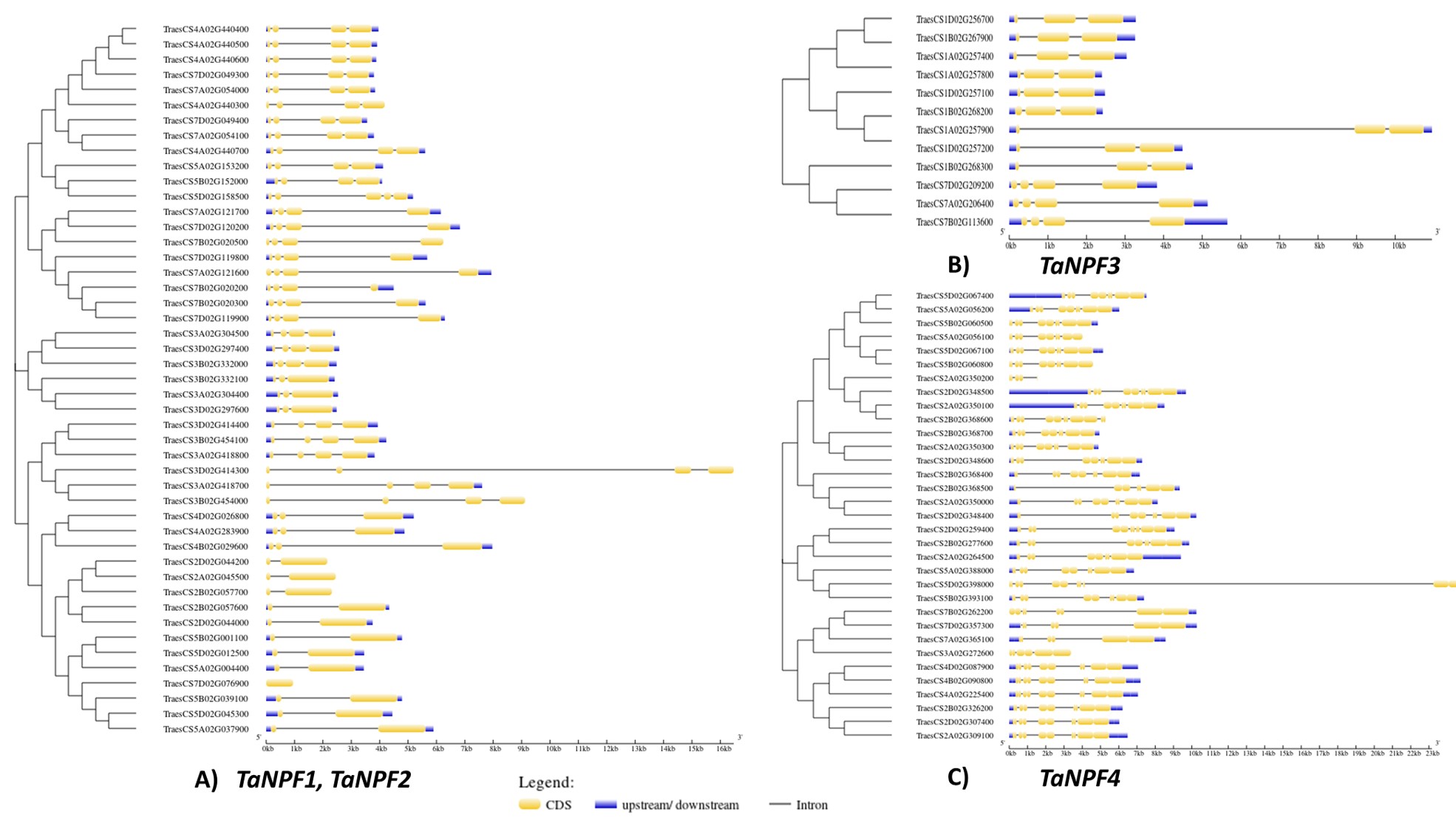


**Supplementary Figure 1a** Gene structures of nitrate transporter genes. **A-** *TaNPF1, TaNPF2,* **B-** *TaNPF3* **C**- *TaNPF4.* Figure was developed by Gene Structure Display Server 2.0 (http://gsds.gao-lab.org/ )(Hu et al 2015).


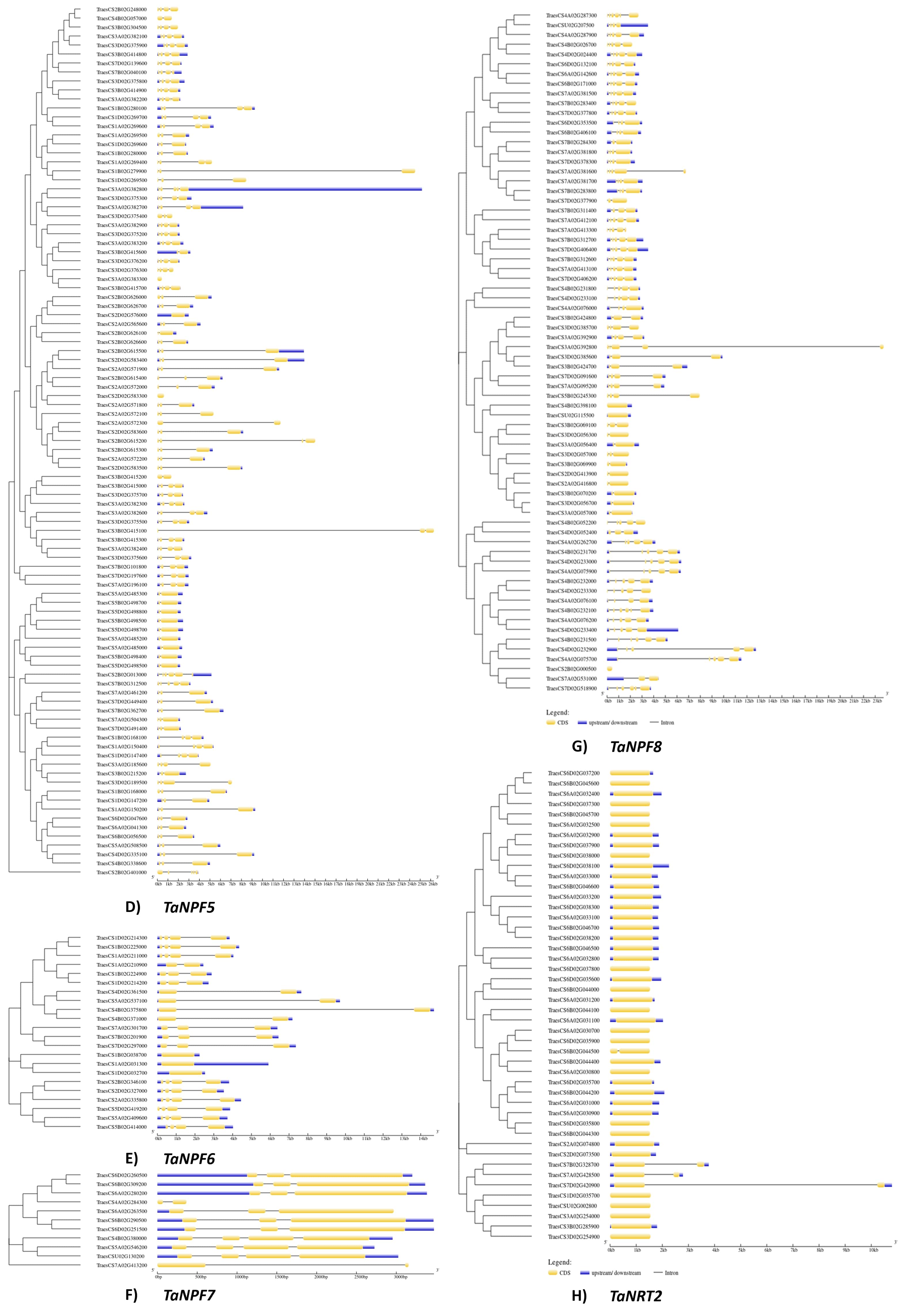


**Supplementary Figure 1b** Gene structures of nitrate transporter genes. **D-** *TaNPF5,* **E-** *TaNPF6* **F**- *TaNPF7,* **G-** *TaNPF8,*  **H-** *TaNRT2. .* Figure was developed by Gene Structure Display Server 2.0 (http://gsds.gao-lab.org/ )(Hu et al 2015).


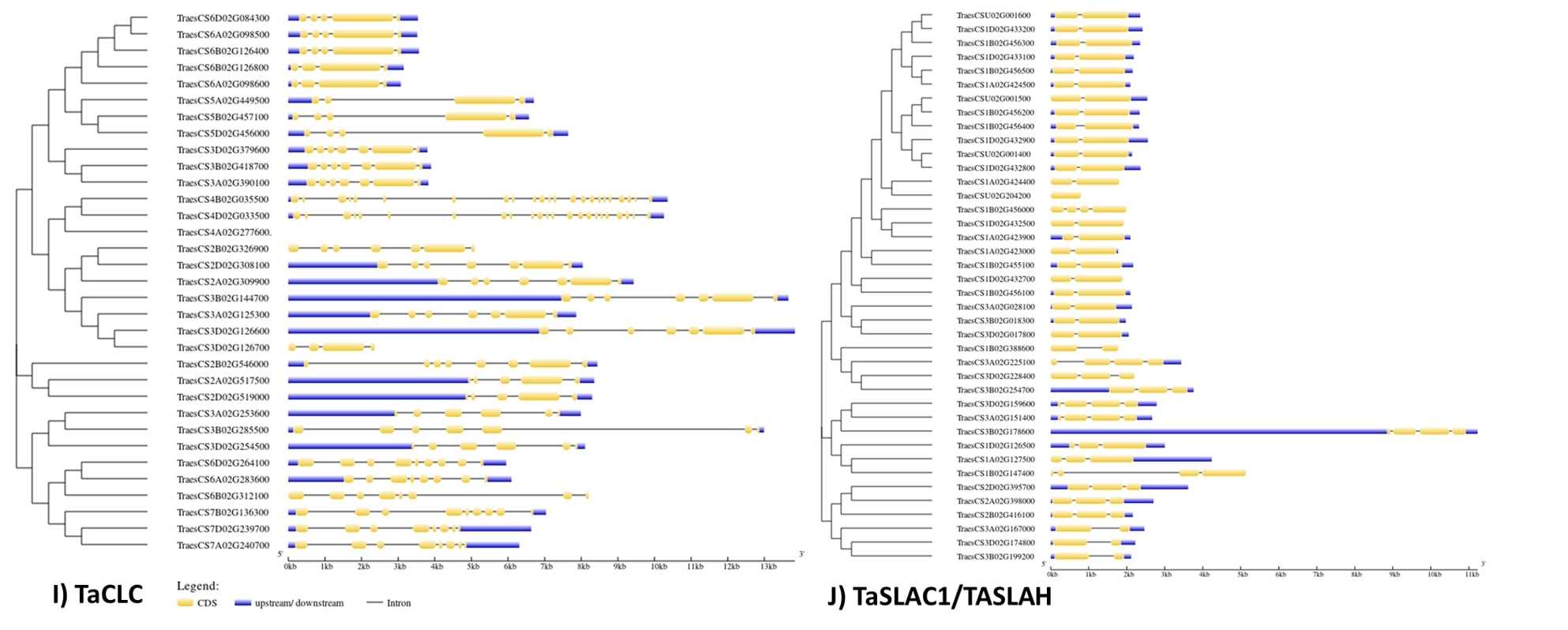


**Supplementary Figure 1c** Gene structures of nitrate transporter genes. **I**-*TaCLC*, **J**- *TaSLAC/TaSLAH.* Figure was developed by Gene Structure Display Server 2.0 (http://gsds.gao-lab.org/) (Hu et al 2015).


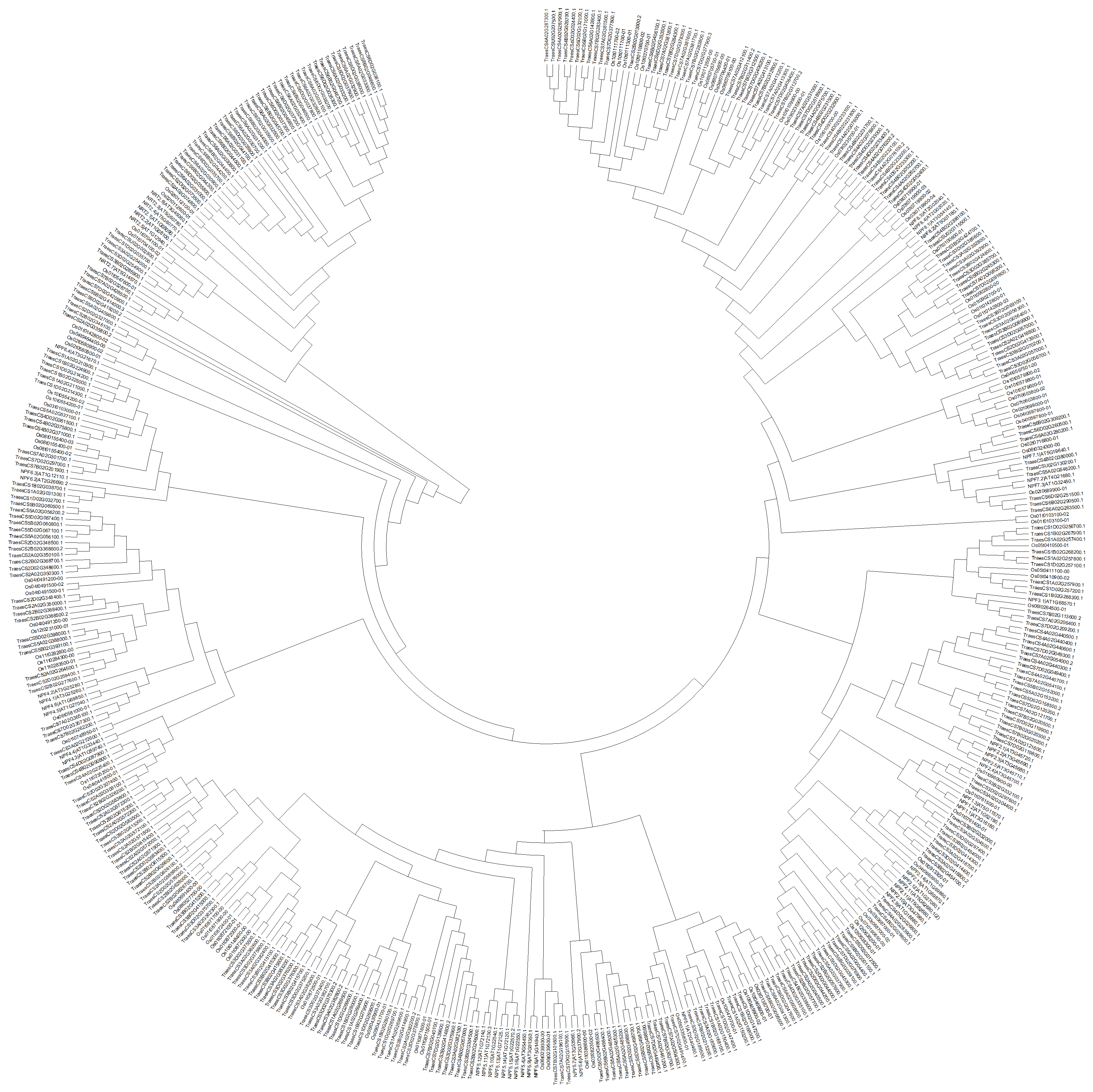


**Supplementary Figure 2** Phylogenetic relationship of wheat NPF and NRT2 genes with Arabidopsis and rice nitrate transporter genes. Figure was developed by MEGA X software (Kumar et al 2018).


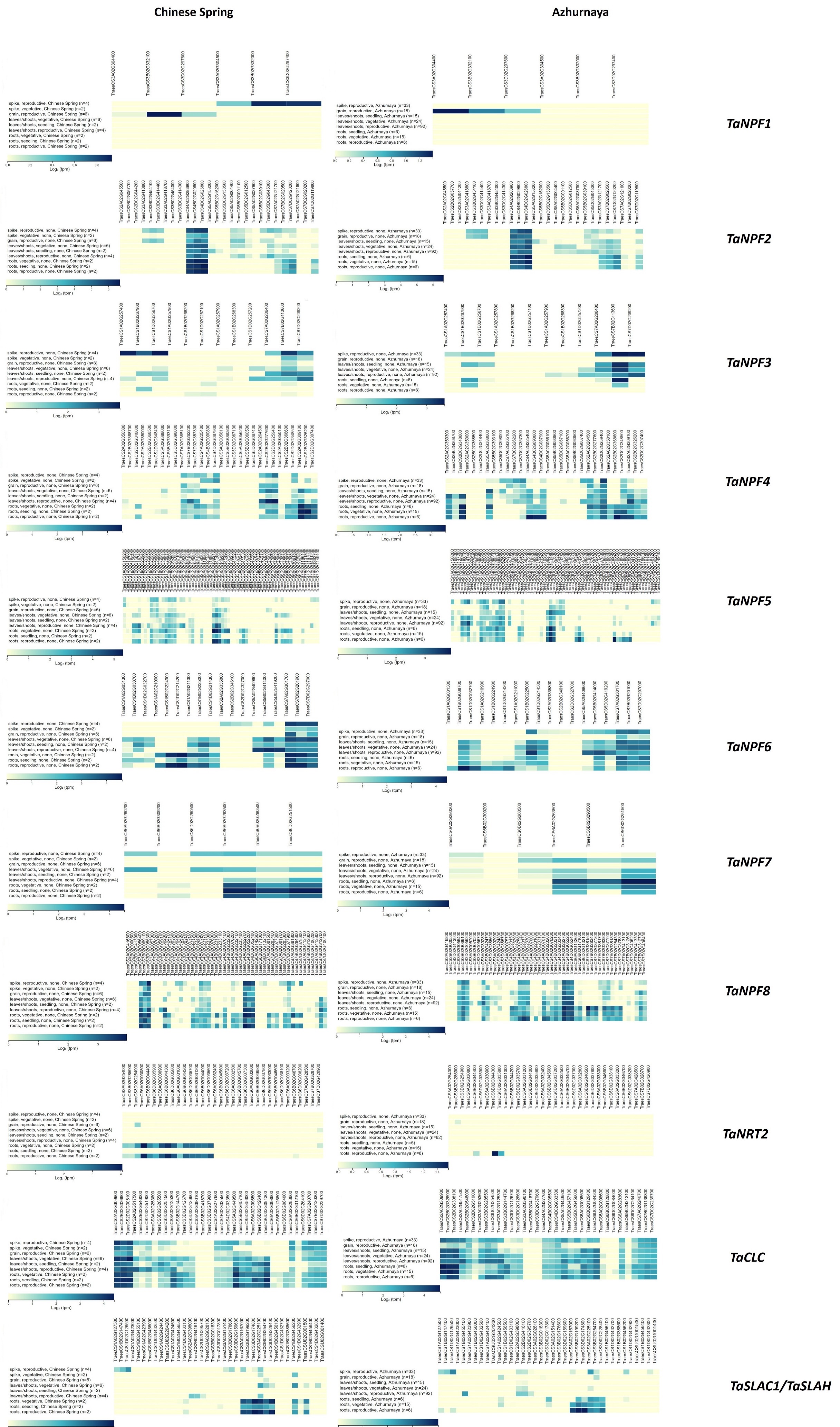


**Supplementary Figure 3** Expression patterns of all nitrate transporter genes in developmental stages of Chinese spring and Azhurnaya. The heat maps were generated by heatmap tool from wheat expression database (http://wheat-expression.com/).


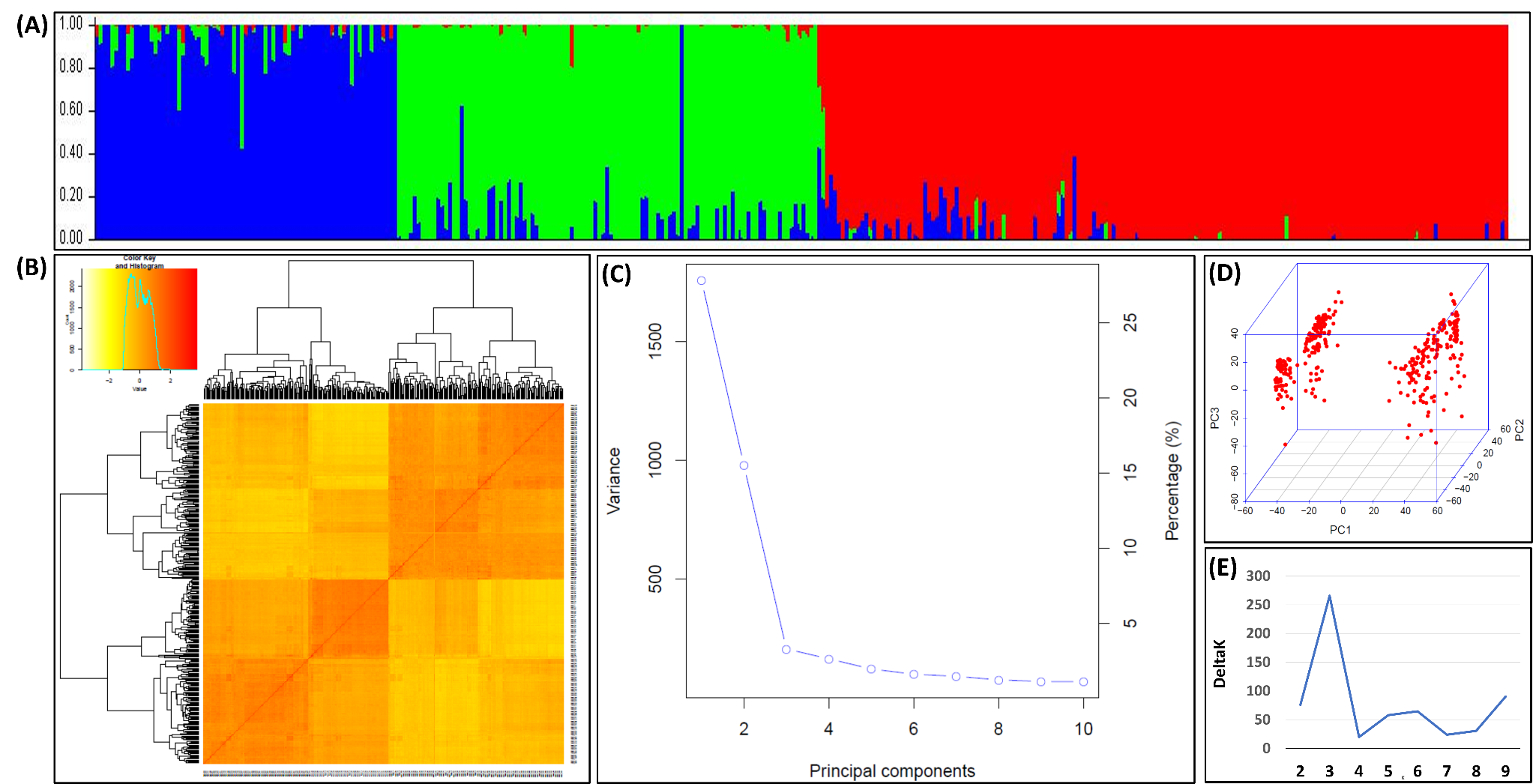


**Supplementary Figure 4 A)** Population structure within the nested synthetic wheat introgression libraries (Sandhu *et al* 2021). **B)** The Kinship matrix displayed as the heat map **C)** The Scree plot indicating the most of the variabilities explained by the first three principal components (PCs) for association study **D)** The genotypic variation among breeding lines constituting the introgression libraries **E)** The appropriate number of the subpopulations determined from the largest delta, *K* = 3. (Sandhu et al 2021).


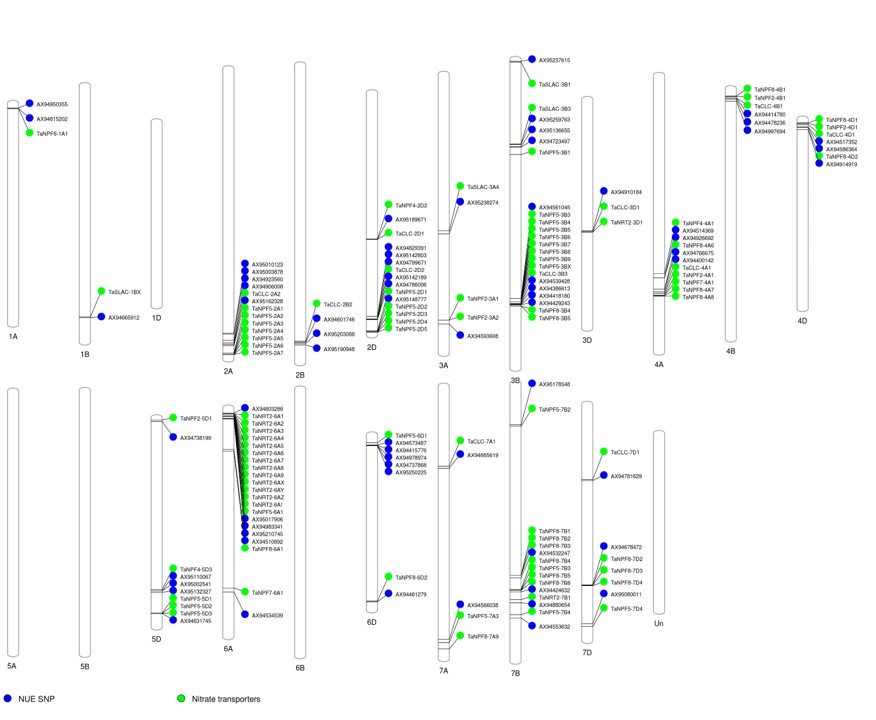


**Supplementary Figure 5** Proximity of nitrogen use efficiency (NUE) linked SNPs to nitrate transporters detected in this study. Figure was generated by web-based software tool Phenogram from Ritchie Lab (http://visualization.ritchielab.org/phenograms/plot).


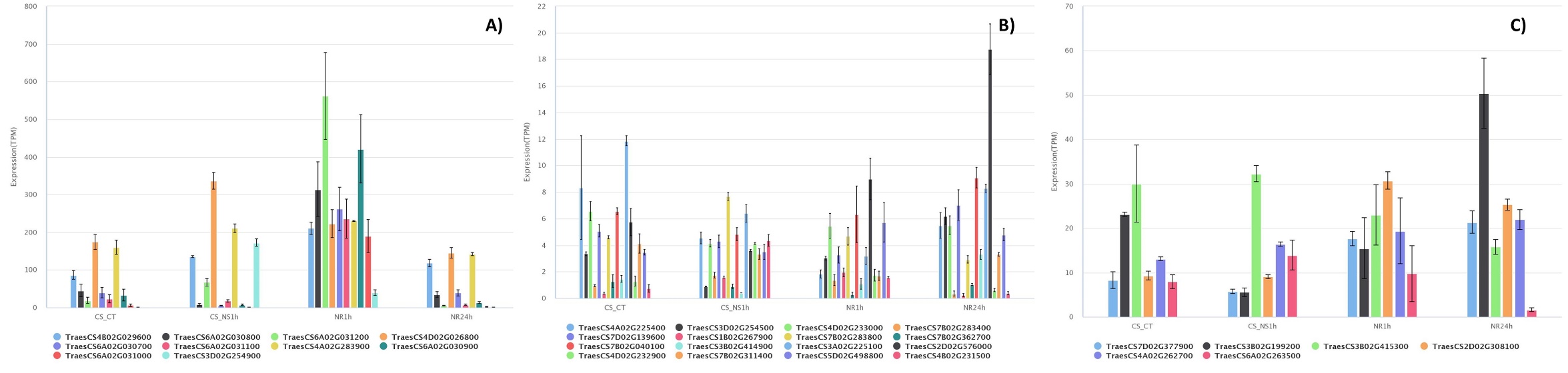


**Supplementary Figure 6:** Expression pattern of selected 32 genes in response to N starvation and N recovery A) Genes expressing at TPM>100 in at least one treatment or control B) Genes expressing at TPM 1-20 C) Genes expressing at TPM 20-100. The graphs were generated by GeneExpression tool from WheatOmics 1.0 database (Ma *et al* 2021)
